# Structure-Related Evolution of Magnetic Order in Anisidinium Tetrachlorocuprates(II)

**DOI:** 10.1021/acs.cgd.3c00066

**Published:** 2023-05-09

**Authors:** Edi Topić, Pavla Šenjug, Dario Barišić, Ivor Lončarić, Damir Pajić, Mirta Rubčić

**Affiliations:** † Department of Chemistry, Faculty of Science, University of Zagreb, Horvatovac 102a, Zagreb 10000, Croatia; ‡ Department of Physics, Faculty of Science, University of Zagreb, Bijenička cesta 32, Zagreb 10000, Croatia; § Ruđer Bošković Institute, Bijenička cesta 54, Zagreb 10000, Croatia

## Abstract

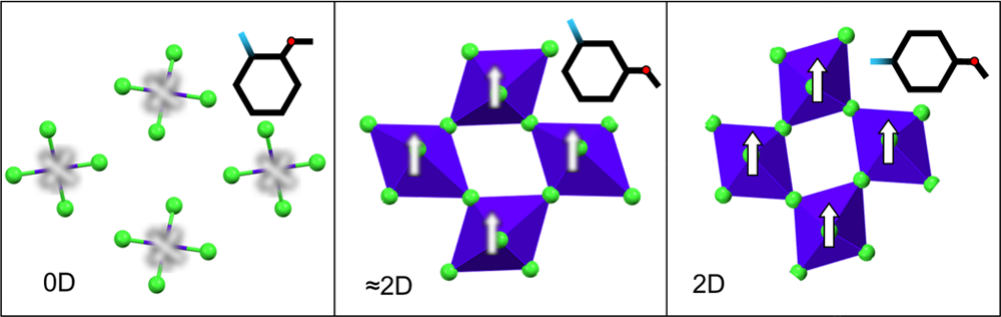

Tetrachlorocuprate(II) hybrids of the three anisidine isomers (*ortho*-, *meta*-, and *para*-, or 2-, 3-, and 4-methoxyaniline, respectively) were prepared and studied in the solid state via X-ray diffraction and magnetization measurements. Depending on the position of the methoxy group of the organic cation, and subsequently, the overall cation geometry, a layered, defective layered, and the structure comprising discrete tetrachlorocuprate(II) units were obtained for the *para*-, *meta*-, and *ortho*-anisidinium hybrids, respectively. In the case of layered and defective layered structures, this affords quasi-2D-layered magnets, demonstrating a complex interplay of strong and weak magnetic interactions that lead to the long-range ferromagnetic (FM) order. In the case of the structure with discrete CuCl_4_^2–^ ions, a peculiar antiferromagnetic (AFM) behavior was revealed. The structural and electronic origins of magnetism are discussed in detail. To supplement it, the method for calculation of dimensionality of the inorganic framework as a function of interaction length was developed. The same was used to discriminate between *n*-dimensional and “almost” *n*-dimensional frameworks, to estimate the organic cation geometry limits for layered halometallates, and to provide additional reasoning behind the observed relation between cation geometry and framework dimensionality, as well as their relation to differences in magnetic behavior.

## Abstract 2

Structural and magnetic consequences of organic cation geometry are evaluated on a series of hybrid organic−inorganic tetrachlorocuprates(II).

## Introduction

In recent years, hybrid organic–inorganic halometallates (HOIHs) have been the protagonists of many cutting-edge studies, ranging from those in the field of optoelectronics to the ones related to complex spintronic nanodevices.^1−7^ Benefits of the HOIH materials with respect to multifunctional but compositionally isotropic ones, such as graphene or MoS_2_, evolve from the fact that HOIHs unify two intertwined sublattices, i.e., organic and inorganic ones, typically only weakly coupled through hydrogen bonding and electrostatic interactions. Each sublattice has its own features; while the inorganic one can give rise to, e.g., magnetic order,^8,9^ organic one can be electrically ordered,^10−13^ and if simultaneously achieved, the two can result in materials with multiple orders.^14−17^ By the proper choice of the building blocks (organic cations, metal cations, and halide anions), diverse structures can be achieved, depending on the spatial arrangement of the halometallate units: three-dimensional (3D), two-dimensional (2D), one-dimensional (1D), and zero-dimensional (0D) structures, each having unique properties with a palpable potential for different applications.^18−20^

The most developed class of HOIHs is 3D ones having the general formula ABX_3_ (A^+^ = organic cation; B^2+^ = inorganic cation; X^–^ = halogen atom), with their structures consisting of a 3D framework of corner-sharing metal halide octahedra, where A^+^ cations are anchored in the framework voids. However, following Goldschmidt’s rule, only cations of an appropriate size can populate such voids and stabilize the resulting assembly, making structural and compositional prospects within this subclass of HOIHs limited.^21^ In contrast, low-dimensional (2D, 1D, and 0D) HOIHs are geometrically less restricted and offer wide compositional and structural prospects for tailoring functional materials. Subsequently, low-dimensional HOIHs are increasingly emerging as a promising class of multifunctional materials, replacing their compositionally and structurally constrained 3D relatives.^22−26^

Low-dimensional HOIHs in the context of magnetism show immense potential.^27−30^ For example, it was recognized that the incorporation of different organic cations or halogen ions in 2D HOIHs will yield different, and in most cases, anisotropic magnetic coupling strengths, resulting in complex magnetic ground states, and potentially nonlinear responses to external stimuli.^31−33^ Moreover, phenomena such as chiral ferromagnetism or multiferroicity have been demonstrated for several 2D HOIHs,^34−37^ which open novel applicative promises for these materials, as well as approaches toward the understanding of fundamental questions of magnetic order in (quasi)two-dimensional systems. However, to be able to truly understand the consequences of structural features on the magnetic properties of the material, it is of paramount importance to isolate and understand which structural parameters are responsible for particular magnetic interactions. Such knowledge should enable in return the development of targeted magnetic HOIHs through judicious selection of organic and inorganic building blocks which can deliver desired geometrical features. One of the significant contributions in this context offers the 2D Perovskites Database^38^ that compares a selection of geometrical parameters for 2D HOIHs and related structures with a limited set of properties (optical and electrical). Another important contribution to the structural systematics of 2D HOIHs is given by McNulty and Lightfoot for an extensive set of haloplumbate hybrids.^39^ However, little information is given regarding the organic cation geometry and how it relates to the geometry of the overall assembly, for which we believe is essential for the future development of materials of this type. Another interesting and quite promising approach utilizes machine learning to predict the possibility of forming 2D HOIH from a set of organic amines.^40^ This approach surely can answer how to make a 2D HOIH, but not necessarily why does the particular organic component construct a specific framework.

Herein, we report a detailed study focusing on the relation between the structure and magnetism in a series of low-dimensional tetrachlorocuprate(II) HOIHs based on the anisidine isomers. Namely, we examine the impact of the geometrical parameters of the building blocks on the dimensionality of the structure and the resulting magnetic behavior. Finally, we present a simple geometrical model connecting the organic cation geometry with the expected dimensionality of the inorganic framework.

## Experimental Section

### Synthesis of Compounds

1 mmol (123 mg) of *ortho-*anisidine or *para-*anisidine free base (sourced from Sigma-Aldrich) was slowly added to a solution of 5 mmol (852 mg) of copper chloride dihydrate (Kemika, Croatia) in 6 mL of 2 M hydrochloric acid. Upon standing (usually within a week) at room temperature, single crystals of **(*o*-A)_2_CuCl_4_** or **(*p*-A)_2_CuCl_4_** precipitate out of the solution. Crystals were isolated by filtration, dried, and stored under dry conditions.

Crystallization attempts for **(*m*-A)_2_CuCl_4_** under the abovementioned conditions yielded also single crystals of partially chlorinated compound ((*m*-A*)_2_CuCl_4_, 4-chloro-3-methoxyanilinium tetrachlorocuprate; structural details are presented in the Supporting Information, Table S1), likely due to very high nucleophilicity of the 4-position on the *m*-anisidinium cation, and the interaction with atmospheric/dissolved oxygen. The tendency of partial chlorination appears to be so high, that the pure nonchlorinated **(*m*-A)_2_CuCl_4_** could be obtained only as a microcrystalline powder from fast crystallization experiment, which was conducted by using 2 mL of concentrated hydrochloric acid instead of 6 mL of 2 M hydrochloric acid. Upon addition of *m-*anisidine to copper chloride/hydrochloric acid solution, **(*m*-A)_2_CuCl_4_** precipitated and was immediately isolated using vacuum filtration and thoroughly dried and stored under dry conditions.

All three compounds are bench-stable once isolated.

### Thermogravimetric Analysis and FTIR Spectroscopy

Thermogravimetric (TG) analysis was carried out with a Mettler-Toledo TGA/SDTA851e thermobalance using aluminum crucibles. All experiments were performed in a dynamic oxygen atmosphere with a flow rate of 200 cm^3^ min^–1^. Heating rates of 5 K min^–1^ were used for all investigations. ATR (attenuated total reflectance) Fourier Transform Infrared spectra (FT-IR) were collected with a Perkin-Elmer Spectrum Two spectrophotometer in the spectral range 4500–450 cm^–1^. Results of the analysis were used for identification purposes and to assess thermal stability and are listed in Supporting Information, Figures S1–S4.

### Single-Crystal X-ray Diffraction (SC-XRD)

Experimental data and the results of structural model refinement are presented in Supporting Information, Table S1 (for (*m*-A*)_2_CuCl_4_) and Table S2 (for **(*o*-A)_2_CuCl_4_** and **(*p*-A)_2_CuCl_4_**), and the most important geometrical parameters of the crystal structure are listed in Table 1. The data for all structures were collected via ω-scans on an Oxford Xcalibur diffractometer equipped with a 4-circle kappa geometry goniometer, CCD Sapphire 3 detector, and graphite-monochromated Mo K_α_ radiation (λ = 0.71073 Å) at 298(2) K. The data processing was performed using the CrysAlisPro software package.^41^ The structures were solved with dual space methods using SHELXT.^42^ The refinement procedure by full-matrix least-squares methods based on *F*^2^ values against all reflections included anisotropic displacement parameters for all non-H atoms. Hydrogen atoms attached to carbons were placed in geometrically idealized positions and were refined using the riding model with *U*_iso_ = 1.2*U*_eq_ of the connected carbon atom or as ideal CH_3_ groups with *U*_iso_ = 1.5*U*_eq_. Hydrogen atoms attached to heteroatoms were located in difference Fourier maps at the final stages of the refinement procedure. Their coordinates were refined freely but with restrained N–H distances of 0.86(2) Å. All refinements were performed using SHELXL.^43^ The SHELX programs operated within the Olex2 suite.^44^ Geometrical calculations and molecular graphics were done with Vesta.^45^ CCDC 2232924–2232927 contain the supplementary crystallographic data for this paper. These data can be obtained free of charge via http://www.ccdc.cam.ac.uk/conts/retrieving.html (or from the Cambridge Crystallographic Data Centre, 12, Union Road, Cambridge CB2 1EZ, UK; fax: +44 1223 336033).

**Table 1:**
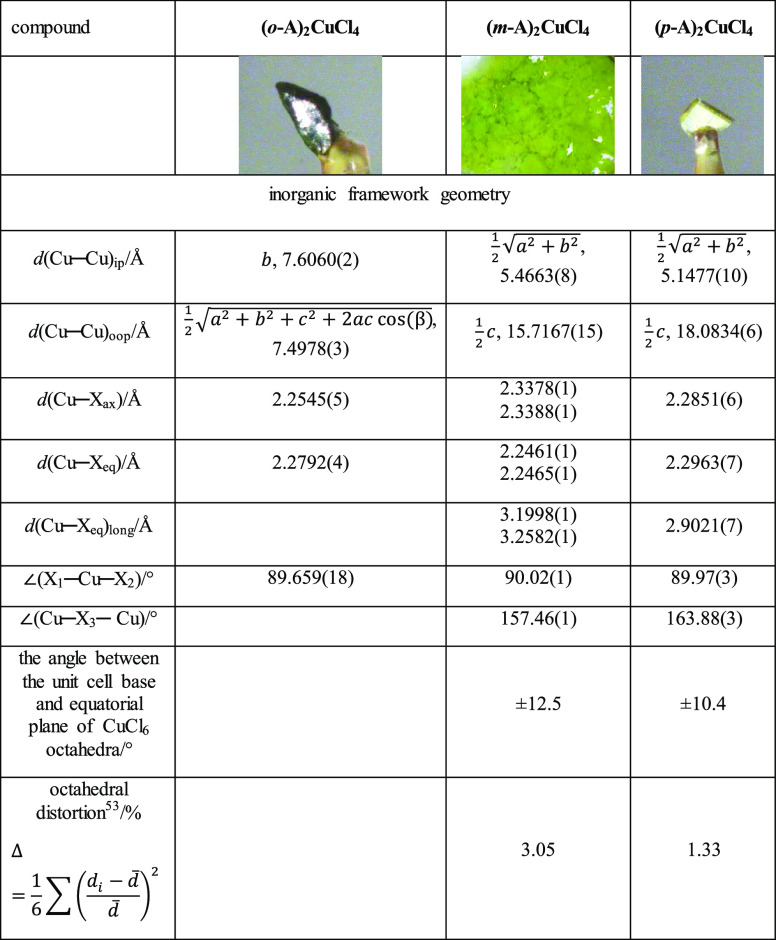
Selected Structural and Geometrical Parameters of Crystal Structures^a^

a Standard deviations of parameters obtained from Rietveld refinement are dependent on the number of simultaneously refined parameters and are usually underestimated.

### Powder X-ray Diffraction (PXRD)

PXRD analyses of bulk **(*o*-A)_2_CuCl_4_** and **(*p*-A)_2_CuCl_4_** samples were performed on a Panalytical Aeris diffractometer in the Bragg–Brentano mode (Supporting Information, Figure S5).

The structural model of **(*m*-A)_2_CuCl_4_** was elucidated from X-ray powder diffraction data collected on a Panalytical Empyrean diffractometer equipped with monochromated (Ge⟨111⟩) Cu Kα_1_ source and a PixCEL HPC detector in the capillary transmission mode. Indexing of the diffraction peaks was done using the N-TREOR program operating in EXPO2014 software.^46^ Rietveld refinement was done in TOPAS Academic software v.5.^47^ Background scattering intensities were modeled using the Chebyshev function over the whole measured 2θ range. Instrument-related and sample-related peak convolution parameters were refined using the fundamental parameters approach coupled with le Bail refinement.^48^ The crystal structure of (*m*-A*)_2_CuCl_4_, with chlorine atoms removed was expanded to the *P*2_1_/*c* subgroup and linearly transformed to a unit cell found by indexation of **(*m*-A)_2_CuCl_4_** powder data. The resulting structure was optimized with periodic density functional theory (pDFT) and used as a starting model and as input regarding fragment constraints. The pDFT calculations were performed using PBE + D3 exchange–correlation functional and medium SG15 basis set as implemented in QuantumATK.^49^ Positions and orientations of two symmetrically independent rigid fragments of *meta*-anisidinum cations and one square-planar tetrachlorocuprate anion were found using a simulated annealing algorithm. Additionally, three independent isotropic displacement parameters were assigned to copper atoms, chlorine atoms, and nonhydrogen atoms of organic fragments, respectively. Displacement parameters of hydrogen atoms were fixed at 1.5 and 1.2 times higher than those of parent carbon and nitrogen atoms, respectively. Powder data showed significant anisotropic peak broadening, which were modeled by the spherical harmonics approach,^50^ Stephens *hkl*-dependent peak width approach,^51^ and with stacking faults along the *c-*direction, with the first two approaches giving almost the same results, and the stacking fault approach giving an inferior fit. Nevertheless, all three results indicate the presence of defects perpendicular to inorganic layers. Detailed results of fits are presented in Supporting Information, Figures S6–S9 and Table S3, and the final stage input files for refinement in TOPAS can be found in Supporting Information, section Powder X-ray diffraction—TOPAS input files.

### Magnetic Measurements

Magnetization measurements were performed on the powders of **(*o*-A)_2_CuCl_4_**, **(*m*-A)_2_CuCl_4_**, and **(*p*-A)_2_CuCl_4_** using a SQUID MPMS 5 magnetometer. The temperature dependence of static magnetization was measured two times from 2 to 300 K, first when the sample was cooled in the zero field and measured while heating in the applied field (ZFC curve). After that, the sample was cooled down in the same applied field and again measured while heating (FC curve). The field dependence of isothermal magnetization was measured at constant temperatures in fields up to 50 kOe.

## Results and Discussion

**Figure 1:**
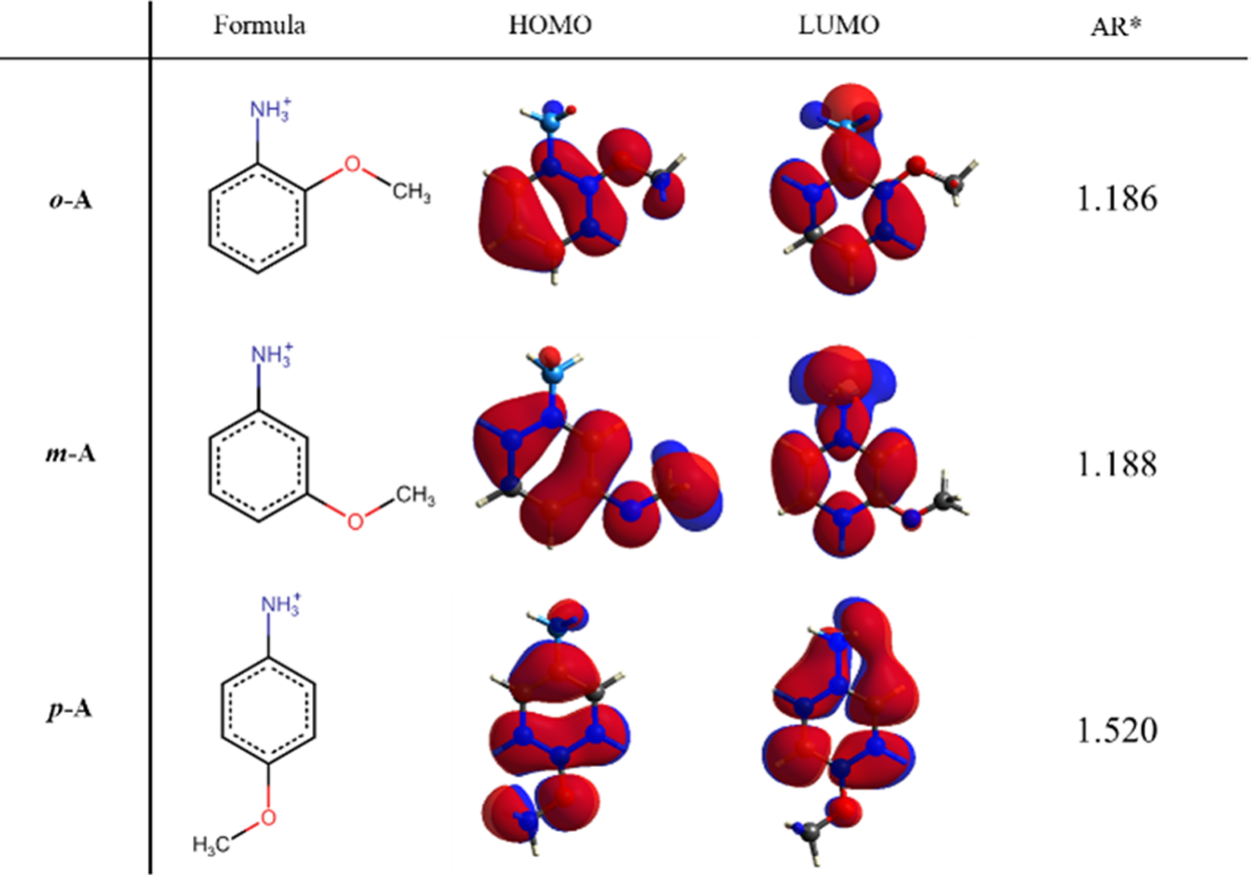
Structural formula, HOMO and LUMO isosurface, and aspect ratios (ARs) of three structural isomers of anisidinium cation, presenting differences in geometrical and electronic structures. Isosurfaces were generated using Tonto quantum chemistry package^52^ (B3LYP/G6311(d,p)). The aspect ratio was calculated for van der Waals bounding box major face.

Anisidine (methoxyaniline) has three constitutional isomers: *ortho-*, *meta-*, and *para-*anisidine, or 2-, 3-, and 4-methoxyaniline, respectively. Anisidine isomers, as well as their cations, have significantly different geometries (Figure 1). Due to the relatively bulky nature of the methoxy group compared to the total size of the molecules/cations, it is anticipated that efficiency in crystal packing will increase from *ortho-*anisidinium to *para*-anisidinium salts. On the other hand, the electron-donating property of the methoxy group coupled with an already electron-rich anilinium ring modulates the electronic structure of the cations depending on the relative position of the ancillary group (Figure 1). Thus, it can be expected that materials derived from anisidinium isomers will not only have significantly different crystal structures but will likely have different magnetic ordering as well. To explore this scenario, tetrachlorocuprate(II) salts of the three anisidine isomers were prepared by crystallization from an aqueous solution containing anisidine, copper chloride, and hydrochloric acid in a molar ratio of 1:5:10, respectively.

The appearance of the crystals of the three synthesized compounds was the first indication of their diverse underlying structures. Namely, *ortho*-anisidinium tetrachlorocuprate(II) (**(*o-*A)_2_CuCl_4_**) crystallizes in the form of dark green prisms, *meta*-anisidinium one (**(*m-*A)_2_CuCl_4_**) as thin light green prisms, and *para*-anisidinium (**(*p-*A)_2_CuCl_4_**) in the form of gold leafy plates (Table 1).

The inorganic framework of the **(*o*-A)_2_CuCl_4_** crystal structure comprises discrete tetrachlorocuprate(II) anions positioned at the unit cell vertices and in its center, while the rest of the space is filled by *ortho*-anisidinium cations. The CuCl_4_^2–^ anion is planar and centrosymmetric with respect to the copper ion, but the distances between the copper ion and two symmetrically nonequivalent chlorine ions differ in length by about 1% (Table 1). In the structures that contain the discrete CuCl_4_^2–^ units, such units are more commonly found in a tetrahedral geometry than the square-planar one. However, there are several examples where the CuCl_4_^2–^ units display square-planar geometry, the most relevant being those built from substituted aminopyridinium cations.^54−57^ In this structure, one observes relatively short hydrogen bonds formed by the ammonium group of the organic cation and the chlorides of the tetrachlorocuprate(II) units. The distance between the neighboring magnetically active ions is relatively large (approximately 7.6 Å) in all directions, suggesting that magnetic coupling will be very weak or nonexistent (Table 1).

**Figure 2:**
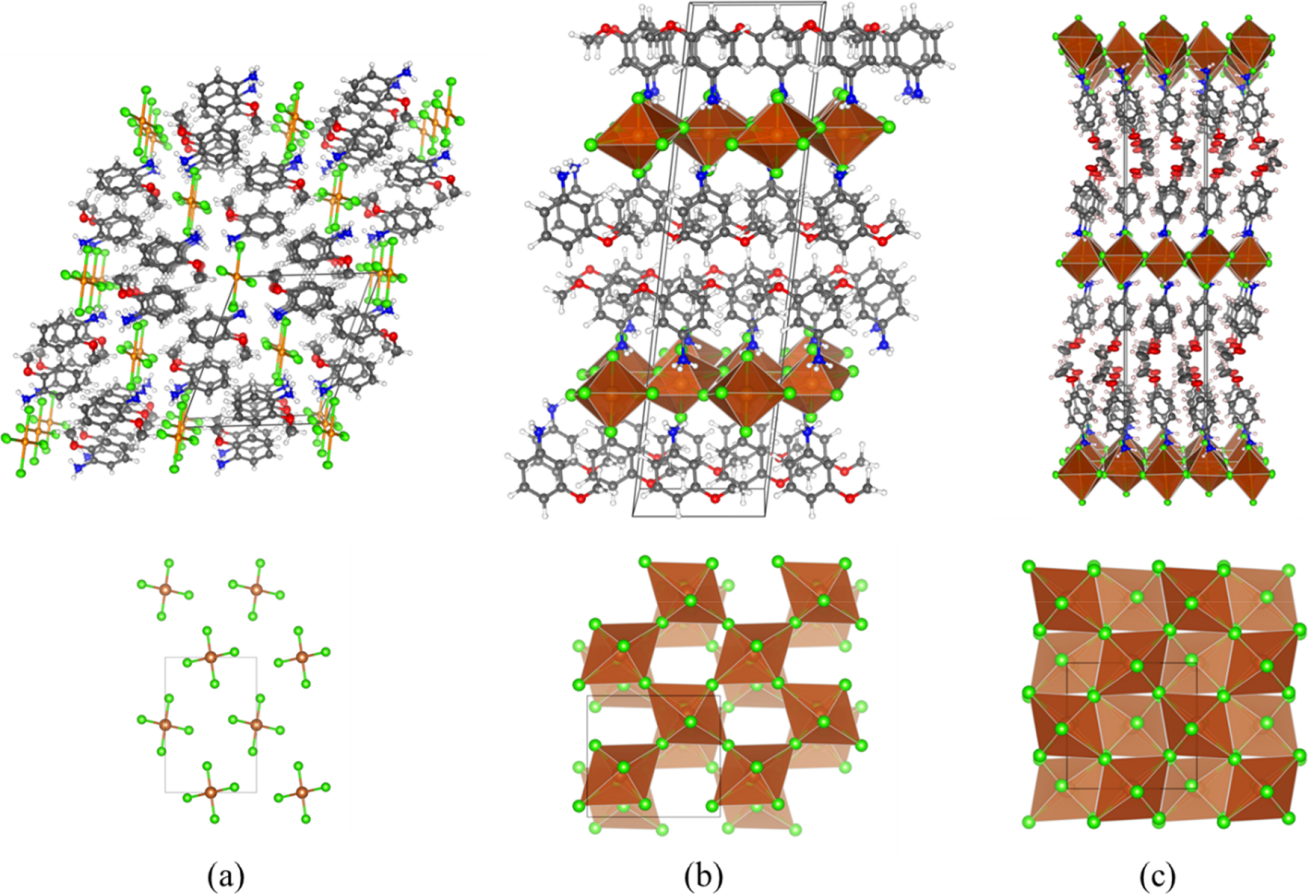
Crystal packing in (a) **(*o*-A)_2_CuCl_4_**, (b) **(*m*-A)_2_CuCl_4_**, and (c) **(*p*-A)_2_CuCl_4_** shown along the crystallographic *b* axis (top) and the crystallographic *c* axis (bottom). In (a) and (c), atoms are shown as displacement ellipsoids at 50% probability. In (b), atoms are shown as spheres of arbitrary radii.

In contrast to **(*o*-A)_2_CuCl_4_**, the structures of **(*m*-A)_2_CuCl_4_** and **(*p*-A)_2_CuCl_4_** can be described as layered (Figure 2), since the distances between copper ions are significantly smaller in two directions (about 5.5 Å) than in the third (>15 Å). Neighboring tetrachlorocuprate(II) ions are rotated by 90° along the direction perpendicular to the formed chlorocuprate(II) planes, so the environment of the copper ion can be described as a highly deformed octahedral (Table 1, parameter Δ; Figure 2). Thus, the inorganic framework can be perceived as consisting of layers of chlorocuprate(II) units, with the deformed octahedral geometry, connected through their vertices. The equatorial planes of the distorted octahedra are not parallel to the base of the unit cell, so the octahedra are tilted in a zigzag fashion (Figure 2).^58^ The potential reason for observing this type of assembly in **(*m*-A)_2_CuCl_4_** and **(*p*-A)_2_CuCl_4_** as opposed to **(*o*-A)_2_CuCl_4_** seemingly stems from the more efficient packaging of organic cations in bilayers due to the more elongated cation geometry (Figure 1, parameter AR). The inorganic framework of the **(*m*-A)_2_CuCl_4_** comprises a slightly less dense tetrachlorocuprate plane (with a larger distance within the plane, Table 1, *d*_ip_) but with a smaller distance between the planes (Table 1, *d*_oop_), while the **(*p*-A)_2_CuCl_4_** forms a denser inorganic plane with a larger gap between the layers. Another important feature of the **(*m*-A)_2_CuCl_4_** is the presence of structural defects, as evidenced by asymmetric peak broadening (for details see Experimental Section and the Supporting Information). This is manifested through slight distortion of periodicity in the *ab* plane, i.e., inorganic planes are not stacked perfectly. From these geometric findings, it can be expected that the coupling between the magnetic ions in the plane will be the strongest in the **(*p*-A)_2_CuCl_4_**, while the coupling between the planes in **(*m*-A)_2_CuCl_4_** and **(*p*-A)_2_CuCl_4_** is not predictable due to the complex interplay between the factors determined by packing, distance, relative positions in space, etc. However, it can be expected that the nature of the magnetic order in **(*m*-A)_2_CuCl_4_** will be influenced by the distorted periodicity.

**Figure 3:**
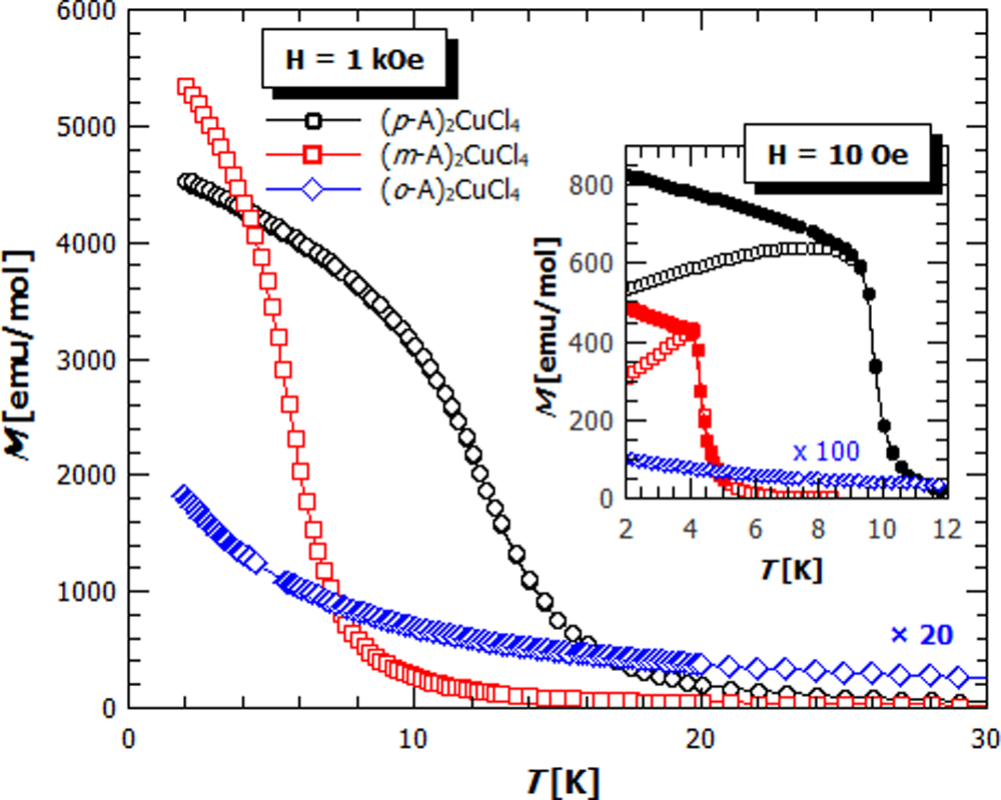
Temperature dependence of magnetization for **(*p*-A)_2_CuCl_4_** (black dots), **(*m*-A)_2_CuCl_4_** (red rectangles), and **(*o*-A)_2_CuCl_4_** (blue diamonds) in the magnetic field of 1 kOe, and 10 Oe (inset: empty symbols—ZFC, full symbols—FC). The magnetization of **(*o*-A)_2_CuCl_4_** was multiplied by factor 20 in *H* = 1 kOe and 100 in *H* = 10 Oe.

**Figure 4:**
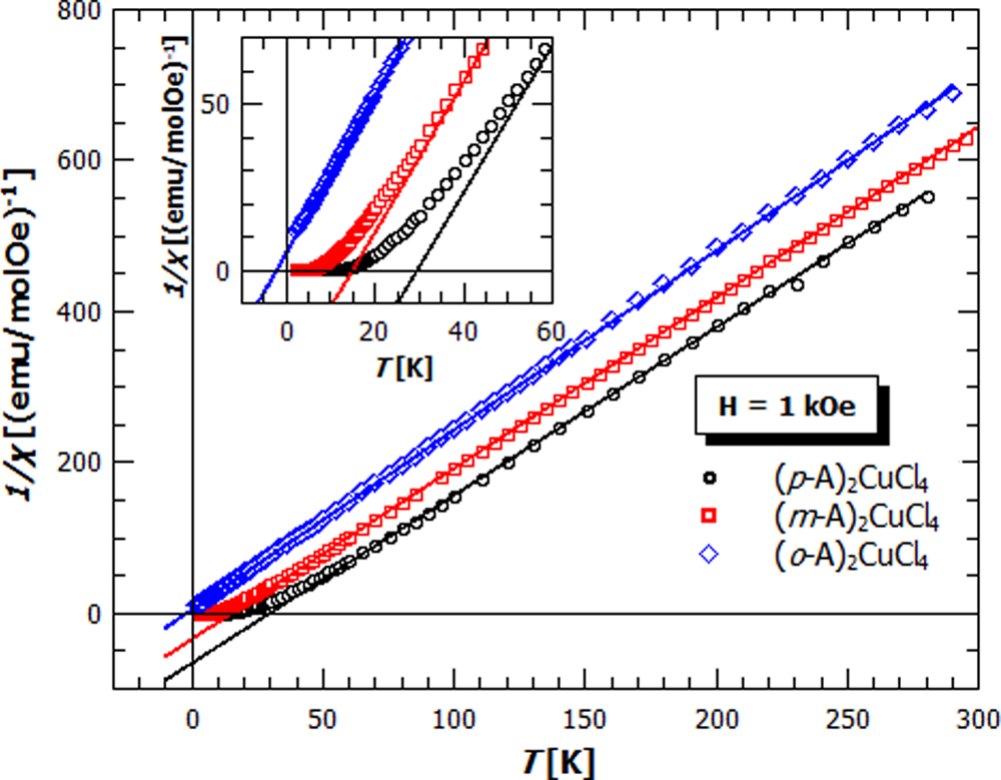
Curie–Weiss fit on the temperature dependence of reciprocal susceptibility of (***p*-A)_2_CuCl_4_** (black dots), **(*m*-A)_2_CuCl_4_** (red rectangles), and **(*o*-A)_2_CuCl_4_** (blue diamonds) in the magnetic field of 1 kOe. Inset: zoom for the lowest temperatures, showing a deviation from the Curie–Weiss law at temperatures much higher than *T*_c_ for **(*m*-A)_2_CuCl_4_** and (***p*-A)_2_CuCl_4_**.

Magnetic interactions and thereby the magnetic behavior for the three compounds were shown to depend greatly on the structure and geometrical parameters. Figure 3 shows the temperature dependence of magnetization in the fields of 1 kOe and 10 Oe (inset). The **(*p*-A)_2_CuCl_4_** and **(*m*-A)_2_CuCl_4_** show a ferromagnetic-like behavior below the transition temperature (*T*_c_) of 9.6 and 4.2 K, respectively, while the **(*o*-A)_2_CuCl_4_**, comprising the discrete CuCl_4_^2–^ units, follows closely the Curie–Weiss law in almost the whole temperature range (Figure 4). The Curie–Weiss fit on the reciprocal susceptibility at high temperatures (Figure 4) gives the *g*-factors in the range of 2.12–2.19, which corresponds well to the values found in the literature for the Cu^2+^ ion, and the Weiss parameter (θ) of 28.7(4), 15.0(2), and −2.8(2) K for the ***p*-**, ***m*-**, and **(*o*-A)_2_CuCl_4_**, respectively. The trend in the Weiss parameters is consistent with the trend of the transition temperatures, with higher θ for the **(*p*-A)_2_CuCl_4_** and lower θ for the **(*m*-A)_2_CuCl_4_**. However, the discrepancies between the ordering temperatures and Weiss parameters, as well as deviation from the Curie–Weiss law well above *T*_c_ (Figure 4, inset) point to the low dimensional nature of magnetic order for **(*m*-A)_2_CuCl_4_** and **(*p*-A)_2_CuCl_4_**. This is typically observed for such quasi-two-dimensional magnetic systems and is also consistent, in our case, with the crystal structures. Considering the dominance of magnetic interactions within the chlorocuprate(II) layers between the four nearest neighbors in **(*m*-A)_2_CuCl_4_** and **(*p*-A)_2_CuCl_4_**, the obtained values of Weiss parameters are a good approximation for the super-exchange *J* parameters between the Cu^2+^ ions. The in-plane ferromagnetic interaction can be explained by considering the orbitals. The Cu^2+^ ions in the elongated octahedral geometry have an unpaired electron in the d_*x*^2^ – *y*^2^_ orbital, and the structure is such that the elongations of the neighboring in-plane octahedra are mutually perpendicular, forming the so-called antiferrodistortive pattern (Supporting Information, Figure S10). This leads to the orthogonal half-filled orbitals and, considering the Goodenough–Kanamori rules, the interaction should be ferromagnetic. Such an ordering is often found in the copper(II)-layered HOIHs.^28,33,59,60^ It can be noted that in **(*m*-A)_2_CuCl_4_**, the angle ∠Cu–X_3_–Cu (157.46°) is further from the 180° than in **(*p*-A)_2_CuCl_4_** (163.88°) which could imply the lower contribution of the antiferromagnetic term in the super-exchange between the nonorthogonal orbitals in **(*m*-A)_2_CuCl_4_**. However, this cannot be simply connected to the total super-exchange which depends also on the distance between the metal ions and the halogen bridge which definitely prevails in the observed increase of the ferromagnetic super-exchange in **(*p*-A)_2_CuCl_4_**. Crucial for the observed efficient ferromagnetic exchange is that the longer metal–halogen distance is arranged in the antiferrodistortive pattern, providing the necessary orbital orthogonality.

**Figure 5:**
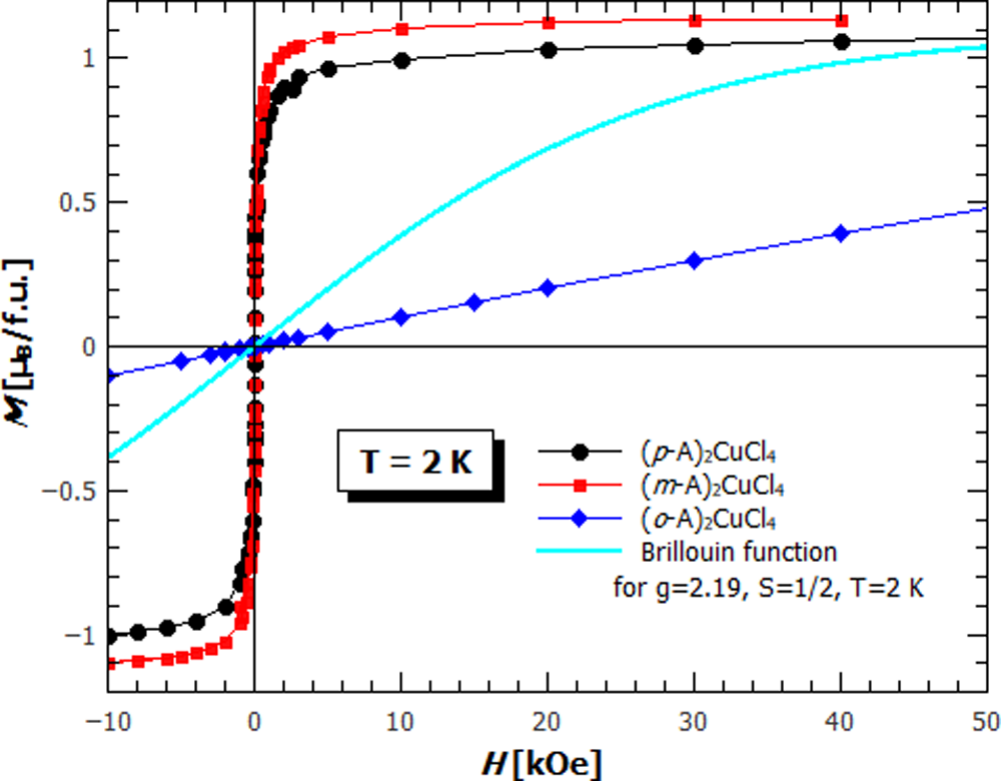
Field dependence of magnetization for **(*p*-A)_2_CuCl_4_** (black dots), **(*m*-A)_2_CuCl_4_** (red rectangles), and **(*o*-A)_2_CuCl_4_** (blue diamonds) at temperature 2 K. The cyan line represents the *M*(*H*) of a paramagnet with a spin 1/2 and *g*-factor 2.19 given by the Brillouin function.

The *M*(*H*) curves of **(*p*-A)_2_CuCl_4_** and **(*m*-A)_2_CuCl_4_** shown in Figure 5 are typical soft ferromagnetic hysteresis with saturation values corresponding to a 1 Cu^2+^ ion per formula unit. The negligible coercive field and very high slope of the *M*(*H*) curve around zero fields support the quasi-two-dimensionality of the systems. Namely, given that magnetic planes are ordered ferromagnetically and reorient as a whole magnetic unit, as well as the fact that much weaker magnetic interaction between the planes allows the easy reorientation of single magnetic planes, there is no pinning which would hinder reorienting so that the reorienting will happen in a relatively weak magnetic field, leading to the observed shape of hysteresis loops.

**Figure 6:**
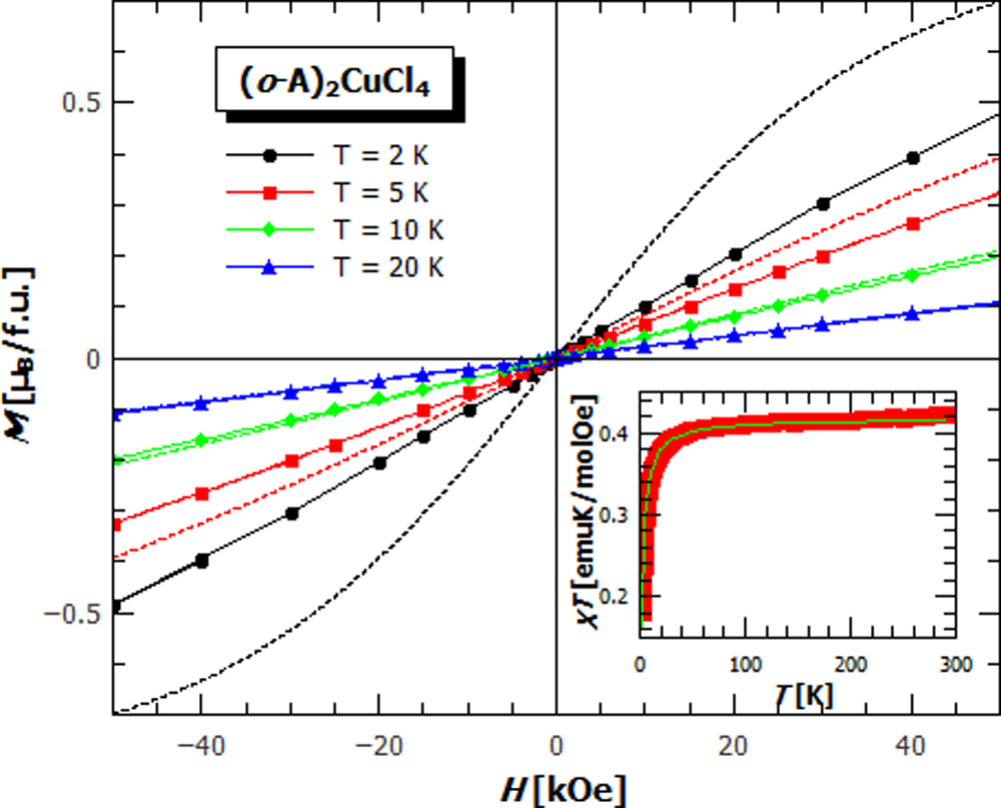
Magnetization measurements for **(*o*-A)_2_CuCl_4_**. Measured *M*(*H*) for different temperatures with respective Brillouin functions (dotted lines). Inset: χ*T*(*T*) with a Bonner–Fisher fit for the antiferromagnetic (AFM) chains of the spin 1/2.

The value of θ for **(*o*-A)_2_CuCl_4_** is small enough (−2.8 K) that the system can be described as paramagnetic in almost the whole measured temperature range with only small deviations below ∼3 K and presumably a magnetic transition below the measurement temperature limit. The presence of magnetic interactions is more strongly expressed in the field dependence of magnetization. Measured *M*(*H*) for **(*o*-A)_2_CuCl_4_** at 2 K clearly deviates from the paramagnetic curve given by the Brillouin function as shown in Figure 5 and follows a linear function, often seen in the antiferromagnets. This also indicates the presence of antiferromagnetic (AFM) interactions and a possible AFM phase transition at the temperatures below 2 K. Figure 6 augments the result of the magnetization measurement: the presence of the AFM interactions in the **(*o*-A)_2_CuCl_4_**, the linear *M*(*H*) curves which do not follow the paramagnetic Brillouin function for the temperatures below 5 K, and χ*T* attaining half of its high-temperature value at 2 K (inset). Considering which interaction could give such a behavior, magnetic dipole–dipole interaction can be excluded due to negligible strength (≈10 mK) as a consequence of large Cu–Cu distances and low spin magnitudes. Evaluating the geometry of hydrogen bonds (Supporting Information, Table S4) that connect neighboring square planar CuCl_4_ units, we have tried to determine whether the 1D AFM chain could be a realistic description of the observed behavior. Fitting the measured data using the Bonner–Fisher model,^61^ where the Hamiltonian is given by *H* = – *J* ∑ *S_i_S_j_*, for the AFM chains of , gave the AFM interaction of *J* = −2.68(5) K. Given some overlap between the anisidinium molecular orbitals exists, the super-exchange interaction could be transferred along other directions and possibly lead to the antiferromagnetic interactions perpendicular to the mentioned chain. The mean-field extension of the used Bonner–Fisher model gave almost the same interactions between the chains as the intra-chain one, but clear conclusions cannot be given due to the lack of lower temperature measurements. This situation is motivating for further research including lower temperatures and ab initio calculations, in order to find out the dimensionality of magnetism and if an exotic magnetic phase exists at very low temperatures. The latter could originate from the competition of weak antiferromagnetic interactions including the hydrogen bond transferred magnetic interaction and super-exchange over the anisidinium molecular orbitals.

Comparing the magnetic results with the crystal structure and relevant geometrical parameters, a correlation is found between the bond lengths and bond angles (Table 1), as well as the phase transition temperature and the Weiss parameter. The latter, which gives the mean interaction between one Cu^2+^ ion and its neighbors, can be taken as an approximation of the strength of in-plane magnetic interactions due to the absence of considerable magnetic interaction between the inorganic planes. Thus, θ^*para*^ > θ^*meta*^ indicates weaker in-plane interactions of **(*m*-A)_2_CuCl_4_** and is explained through its longer Cu–Cu distances in the inorganic layers than in **(*p*-A)_2_CuCl_4_**. The lower *T*_c_ of the **(*m*-A)_2_CuCl_4_** can be comprehended by larger in-plane Cu–Cu distances and higher deformation of Cu–Cl–Cu bridges, and the presence of structural defects. Regarding the long-range magnetic order, these effects surmount the smaller Cu–Cu out-of-plane distance in **(*m*-A)_2_CuCl_4_**.

Observing such a remarkable difference in structural and magnetic properties between materials based on similar building blocks motivated us to seek a generalized, “chemistry-invariant” reasoning behind it. It is evident that by changing the organic cation geometry one can alter the dimensionality of the inorganic framework, and thus its properties but the relation is not that straightforward. From the material design standpoint, it would be beneficial to know: (1) which organic cations are compatible with a given inorganic framework dimensionality, and inversely, (2) for a given organic cation, what is the expected dimensionality of the inorganic framework. In order to address these questions, it was necessary to set a robust definition of dimensionality that easily differentiates between discrete units and *n*D frameworks, and, ideally, discerns the “almost” *n*D frameworks from true *n*D frameworks. For this purpose, we have developed a method for the calculation of dimensionality as a function of the threshold *t_n_*, which is the maximal metal ion–metal ion distance which can build a framework normalized to the expected M–X–M distance (for details, see Supporting Information, section Dimensionality Calculation).

**Figure 7:**
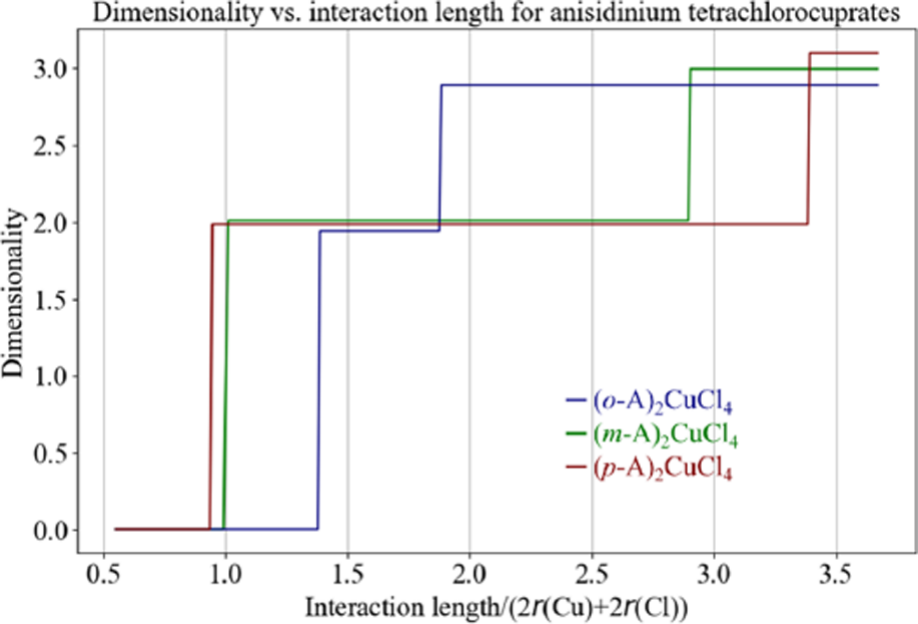
Dimensionality of anisidinium tetrachlorocuprates as a function of interaction length, i.e., the expected Cu–Cu distance (2*r_i_*(Cu) + 2*r_i_*(Cl)).

An illustration of dimensionality calculation, for anisidinium tetrachlorocuprates(II) described herein, is given in Figure 7. Based on these results, the **(*p*-A)_2_CuCl_4_** inorganic framework can be considered truly two-dimensional, as its dimensionality is close to two even at *t_n_* < 1, while its dimensionality becomes close to three only when *t_n_* > 3.38. On the other hand, **(*o*-A)_2_CuCl_4_** contains discrete subunits which would become a 2D framework for *t_n_* > 1.38, or 3D framework for *t_n_* > 1.88. Finally, **(*m*-A)_2_CuCl_4_** is an intermediate case, which becomes 2D at *t_n_* > 1 and 3D at *t_n_* > 2.90.

This approach helps to, at least qualitatively, differentiate between a true 2D HOIH **(*p*-A)_2_CuCl_4_** and its nearly-2D counterpart **(*m*-A)_2_CuCl_4_**, and provides additional perspective regarding the significant difference in magnetic order temperature. Although from the magnetic point of view, such systems are quasi-two-dimensional, in the sense that there are stronger ferromagnetic interactions within the planes, and orders of magnitude weaker interaction between the planes, the origin of perpendicular weak magnetic interactions is still not well understood. Nonetheless, as evidenced by the case of **(*p*-A)_2_CuCl_4_** and **(*m*-A)_2_CuCl_4_**, one can perceive the impact of geometrical dimensionality on magnetic behavior. This is additionally supported by the different temperature dependence of magnetization near the phase transition temperature, where **(*p*-A)_2_CuCl_4_** has a presumably ferromagnetic transition, while **(*m*-A)_2_CuCl_4_** has reminiscence of magnetic frustrations and disorder in the ground state. Moreover, the calculations unveil that the metal centers of **(*o*-A)_2_CuCl_4_** form a 3D lattice at much lower interaction lengths than the other two materials. It can be speculated that such a difference in dimensionality vs interaction length would provoke interesting competition between various long-range interactions, albeit at much lower temperatures.

**Figure 8:**
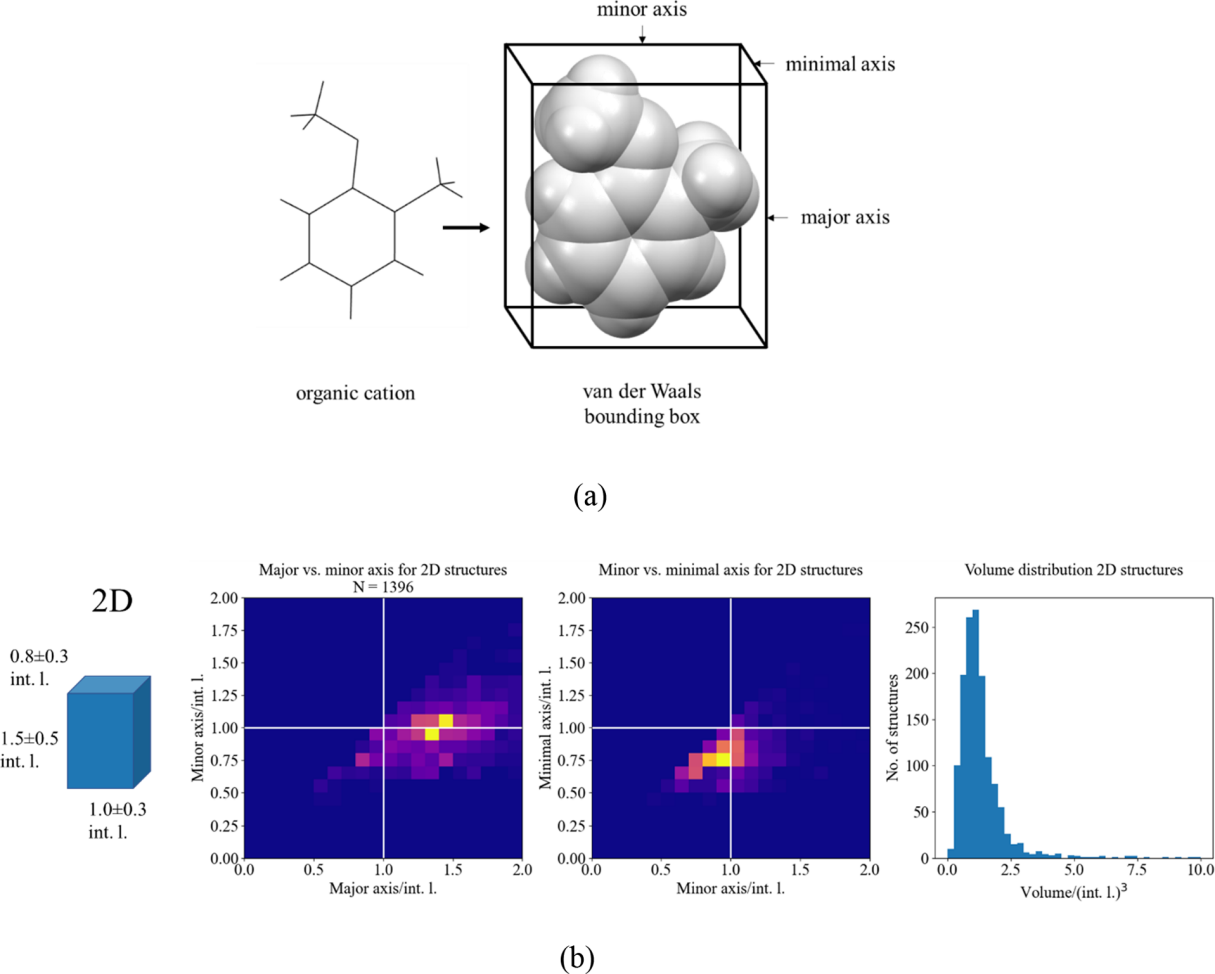
(a) Definition of organic cation van der Waals bounding box parameters. (b) Organic cation bounding box dimension histograms for 2D HOIHs. Figures represent (from left to right): the average bounding box, major vs minor axis distribution, minor vs minimal axis distribution, and volume distribution. All dimensions are normalized to interaction length, i.e., the expected metal–metal distance (2*r_i_*(M) + 2*r_i_*(X)).

Applying the above methodology, one can calculate dimensionality for already reported structures and examine the geometry of organic cations, which can provide information about the geometrical limits of a particular framework dimensionality. To quantify the geometry of organic cations, we have chosen parameters of the van der Waals bounding box, which are one of the simplest descriptors calculable for any molecule or ion. Details of implementation can be found in Supporting Information, section Bounding Box Calculation. When plotted together, the dimensionality and bounding box parameters show the geometrical limits of organic cation for each dimensionality of the inorganic framework (Figure 8 and Supporting Information, Figure S11). This enables a direct comparison of organic cation dimensions with expected metal–metal distances, thus providing a quantitative answer to the question—what organic cations are compatible with *n*D HOIH frameworks.

Structural constraints on organic cations are well known for 3D HOIHs, as they are well established in the context of hybrid perovskites. Apart from an occasional outlier, most of the structures contain an organic cation with a size of less than 1 interaction length (i.e., the expected metal–metal distance, 2*r_i_*(M) + 2*r_i_*(X)) in all directions, which is expected given that (pseudo)cubic voids in 3D HOIHs will be not much larger than the length of M–X–M moiety. Consequentially, most organic cations are less than 1 interaction length cubed in volume (Supporting Information, Figure S11).

**Figure 9:**
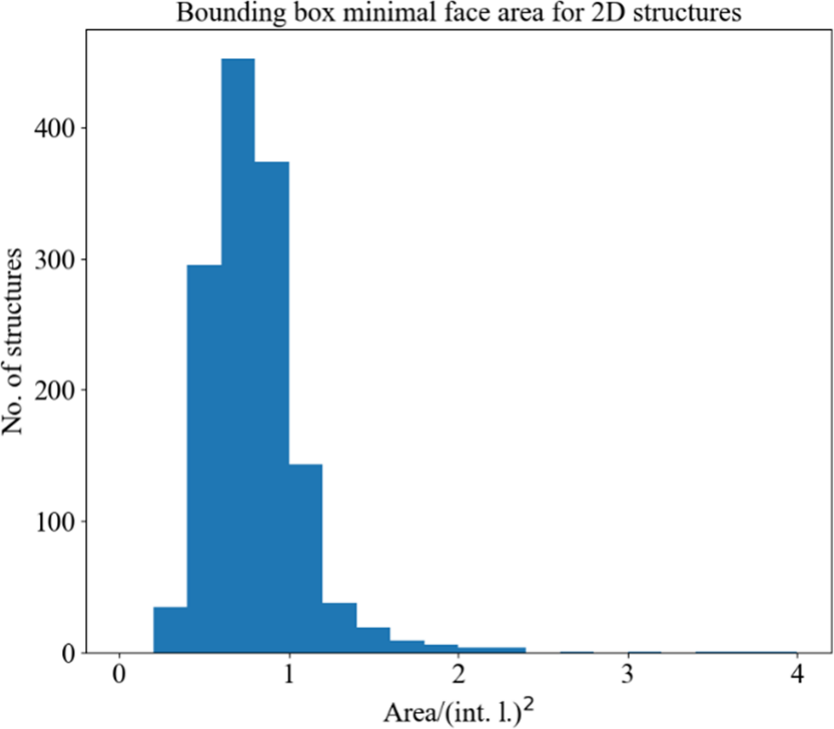
Organic cation bounding box minimal face area distribution for 2D HOIHs. Area is normalized to interaction length, i.e., the expected metal–metal distance (2*r_i_*(M) + 2*r_i_*(X)).

On the other hand, it is apparent that organic cations in 2D HOIHs can be significantly larger, albeit only along the major axis. A lot of examples are known (i.e., long-chain alkylammonium tetrahalometallates) where organic cation is several interaction lengths long (Figure 8). However, the other two dimensions should be such that the cation fits in the space between the four metal centers spanning the lattice base (Supporting Information, Figure S12). In other words, the area of the cation bounding box minimal face should be equal to or less than that of the parallelogram spanned by metal centers, namely one interaction length squared. This is largely supported in observed structures (Figure 9), outlier possibly coming from the overlap of cation bounding boxes in some crystal structures.

The constraints for low-dimensional HOIHs are less obvious, in the sense that no strict limits can be found for bounding box dimensions (Supporting Information, Figure S11). However, it can be concluded that 1D, and particularly discrete systems, can accommodate much larger cations than 2D or 3D HOIHs. Finally, our surface-scratching answer to the first question is (1) small cations can build 3D HOIHs, (2) slim cations can build 2D HOIHs, (3) large cations can build 1D HOIHs, and (4) anything can build HOIHs with discrete inorganic ions. Evidently, some chemical information and possibly geometrical information must be included in this reasoning in order to improve it, for instance, the prevalence of primary ammonium cations as building blocks of 2D HOIHs, and the fact that bounding box methodology overestimates the dimensions for some (flexible) organic cations.

Once geometrical limits are established, one can use them to estimate the dimensionality of the HOIH material derived from a particular organic cation. We have calculated the bounding box parameters for anisidinium cations and compared them to the observed geometrical limits for 2D HOIH frameworks (Table 2). Taking into account the distributions of the minor axis, minimal axis, as well as volume and minimal face area, it can be concluded that the chance of observing a 2D inorganic framework is highest for **(*p*-A)_2_CuCl_4_** where all of the geometric parameters fall well within the limits expected for 2D HOIHs. On the other hand, for **(*m*-A)_2_CuCl_4_**, and particularly for **(*o*-A)_2_CuCl_4_**, the minor axis and minimal face area are slightly above the limits expected for 2D frameworks. Thus, it can be concluded that observation of (1) defects and spin disorder in **(*m*-A)_2_CuCl_4_** and (2) discrete CuCl_4_^2–^ units in the structure of **(*o*-A)_2_CuCl_4_** can be attributed to (1) slight and (2) severe geometrical incompatibility of 2D CuCl_4_ layers with bulkier ***m-*A** and ***o*-A** cations, respectively.

**Table 2:** Organic Cation Bounding Box (OCBB) Dimensions for Anisidinium Tetrachlorocuprates^a^

compound	**(*o*-A)_2_CuCl_4_**	**(*m*-A)_2_CuCl_4_**	**(*p*-A)_2_CuCl_4_**
OCBB major axis/int. l. (Å)^b^	1.696 (9.227)	1.665 (9.060)	1.859 (10.112)
OCBB minor axis/int. l. (Å)^c^	1.430 (7.781)	1.402 (7.627)	1.223 (6.655)
OCBB minimal axis/int. l. (Å)^c^	0.735 (4.000)	0.722 (3.928)	0.729 (3.966)
volume/int. l.^3^ (Å^3^)^b^	1.783 (287.19)	1.686 (271.45)	1.658 (266.94)
minimal face area/int. l.^2^ (Å^3^)^d^	1.051 (31.12)	1.012 (29.96)	0.892 (26.39)

a Interaction length is equal to the expected Cu–Cu distance, i.e., 2*r_i_*(Cu) + 2*r_i_*(Cl) = 5.44 *Å*.

b In principle unlimited for 2D HOIHs.

c No larger than diagonals of parallelogram spanned by metal centers, in this case  int. l.

d No larger than the area of parallelogram spanned by metal centers, in this case 1 int. l.

It must be noted that the abovementioned conclusions depend upon an arbitrary selection of interaction length values, as well as the bounding box calculation method. By extension, the obtained results are a function of selected ionic and van der Waals radii, the quality of crystal structure data, and all the parameters upon which the crystal structure depends on. Thus, the described methodology must be considered only as a naive, one-dimensional starting point in the analysis of HOIH compounds, which could be upgraded for a more complete understanding of these peculiar materials.

## Conclusions

Through the elucidation of structural and magnetic features of anisidinium tetrachlorocuprates, we have demonstrated the superficial geometrical limits of 2D HOIH frameworks, and the remarkable divergence of magnetic behavior in the vicinity of those limits. The connectivity of the inorganic framework and the overall strength of magnetic interactions increases as the anisidinium cation becomes more geometrically compatible with the layered inorganic framework. These results demonstrate that regular packing is an important parameter when considering ways of increasing the magnetic ordering temperature, as evidenced by **(*p*-A)_2_CuCl_4_** and **(*m*-A)_2_CuCl_4_**. Namely, both materials have ferromagnetically ordered planes that couple into the long-range magnetic order, but regularly layered **(*p*-A)_2_CuCl_4_** has a higher magnetic transition temperature than defective layered **(*m*-A)_2_CuCl_4_**, despite the larger distance between magnetic planes. On the other side, **(*o*-A)_2_CuCl_4_** shows the perspective for the class of HOIHs with discrete MX_4_ units, by exploiting the antiferromagnetic interactions, which are generally rare in such metal–organic systems, but important for the emergence of interesting phases and phenomena like spin-liquids, multiferroicity, and magnetoelectric coupling. Finally, the consideration of dimensionality presented herein presents a useful first-principles guideline for the design and structural analysis of HOIHs.

## Supporting Information Available

Experimental and crystallographic data for (***m-*A***)**_2_CuCl**_4_ (Table S1); FTIR spectra (Figure S1); TGA (black) and DTA (red) curves for **(*o*-A)_2_CuCl_4_**,**(*m*-A)_2_CuCl_4_** and **(*p*-A)_2_CuCl_4_** (Figures S2–S4); experimental and crystallographic data for prepared compounds **(*o*-A)_2_CuCl_4_**, **(*m*-A)_2_CuCl_4_** and **(*p*-A)_2_CuCl_4_** (Table S2); measured (black) and calculated (red) PXRD data (Figure S5); results of Rietveld refinement (Figures S6–S8); sum of spherical harmonics obtained by Rietveld refinement (Figure S9); powder data refinement statistics (Table S3); antiferrodistortive arrangement (Figure S10); hydrogen bond geometry in the crystal structures (Table S4); organic cation bounding box dimension histograms (Figure S11); theoretical limits of the organic cation bounding box (Figure S12) (PDF)

## References

[ref1] Babu R.; Giribabu L.; Singh S. P. Recent Advances in Halide-Based Perovskite Crystals and Their Optoelectronic Applications. Cryst. Growth Des. 2018, 18, 2645–2664. 10.1021/acs.cgd.7b01767.

[ref2] Kahwagi R. F.; Thornton S. T.; Smith B.; Koleilat G. I. Dimensionality Engineering of Metal Halide Perovskites. Front. Optoelectron. 2020, 13, 196–224. 10.1007/s12200-020-1039-6. 36641576 PMC9743879

[ref3] Wang J.; Zhang C.; Liu H.; McLaughlin R.; Zhai Y.; Vardeny S. R.; Liu X.; McGill S.; Semenov D.; Guo H.; Tsuchikawa R.; Deshpande V. v.; Sun D.; Vardeny Z. V. Spin-Optoelectronic Devices Based on Hybrid Organic-Inorganic Trihalide Perovskites. Nat. Commun. 2019, 10, 129 10.1038/s41467-018-07952-x. 30631053 PMC6328620

[ref4] Zhai Y.; Baniya S.; Zhang C.; Li J.; Haney P.; Sheng C.-X.; Ehrenfreund E.; Vardeny Z. V. Giant Rashba Splitting in 2D Organic-Inorganic Halide Perovskites Measured by Transient Spectroscopies. Sci. Adv. 2017, 3, e1700704 10.1126/sciadv.1700704. 28782030 PMC5533538

[ref5] Zhao X.; Wang S.; Shan X.; Meng G.; Fang X. Fabrications of Halide Perovskite Single-Crystal Slices and Their Applications in Solar Cells, Photodetectors, and LEDs. Cryst. Growth Des. 2021, 21, 5983–5997. 10.1021/acs.cgd.1c00548.

[ref6] Privitera A.; Righetto M.; Cacialli F.; Riede M. K. Perspectives of Organic and Perovskite-Based Spintronics. Adv. Opt. Mater. 2021, 9, 2100215 10.1002/adom.202100215.

[ref7] Fu Y.; Zhu H.; Chen J.; Hautzinger M. P.; Zhu X.-Y.; Jin S. Metal Halide Perovskite Nanostructures for Optoelectronic Applications and the Study of Physical Properties. Nat. Rev. Mater. 2019, 4, 169–188. 10.1038/s41578-019-0080-9.

[ref8] Asensio Y.; Marras S.; Spirito D.; Gobbi M.; Ipatov M.; Casanova F.; Mateo-Alonso A.; Hueso L. E.; Martín-García B. Magnetic Properties of Layered Hybrid Organic-Inorganic Metal-Halide Perovskites: Transition Metal, Organic Cation and Perovskite Phase Effects. Adv. Funct. Mater. 2022, 32, 2207988 10.1002/adfm.202207988.

[ref9] Ning W.; Gao F. Structural and Functional Diversity in Lead-Free Halide Perovskite Materials. Adv. Mater. 2019, 31, 1900326 10.1002/adma.201900326. 31025419

[ref10] Siwach P.; Sikarwar P.; Halpati J. S.; Chandiran A. K. Design of Above-Room-Temperature Ferroelectric Two-Dimensional Layered Halide Perovskites. J. Mater. Chem. A 2022, 10, 8719–8738. 10.1039/D1TA09537D.

[ref11] Zheng W.; Wang X.; Zhang X.; Chen B.; Suo H.; Xing Z.; Wang Y.; Wei H.; Chen J.; Guo Y.; Wang F. Emerging Halide Perovskite Ferroelectrics. Adv. Mater. 2023, 2205410 10.1002/adma.202205410. 36517207

[ref12] Ambrosio F.; de Angelis F.; Goñi A. R. The Ferroelectric–Ferroelastic Debate about Metal Halide Perovskites. J. Phys. Chem. Lett. 2022, 13, 7731–7740. 10.1021/acs.jpclett.2c01945. 35969174 PMC9421894

[ref13] Jain P.; Stroppa A.; Nabok D.; Marino A.; Rubano A.; Paparo D.; Matsubara M.; Nakotte H.; Fiebig M.; Picozzi S.; Choi E. S.; Cheetham A. K.; Draxl C.; Dalal N. S.; Zapf V. S. Switchable Electric Polarization and Ferroelectric Domains in a Metal-Organic-Framework. NPJ Quantum Mater. 2016, 1, 16012 10.1038/npjquantmats.2016.12.

[ref14] Spaldin N. A.; Ramesh R. Advances in Magnetoelectric Multiferroics. Nat. Mater. 2019, 18, 203–212. 10.1038/s41563-018-0275-2. 30783227

[ref15] Eerenstein W.; Mathur N. D.; Scott J. F. Multiferroic and Magnetoelectric Materials. Nature 2006, 442, 759–765. 10.1038/nature05023. 16915279

[ref16] Cheong S.-W.; Mostovoy M. Multiferroics: A Magnetic Twist for Ferroelectricity. Nat. Mater. 2007, 6, 13–20. 10.1038/nmat1804. 17199121

[ref17] Stroppa A.; Jain P.; Barone P.; Marsman M.; Perez-Mato J. M.; Cheetham A. K.; Kroto H. W.; Picozzi S. Electric Control of Magnetization and Interplay between Orbital Ordering and Ferroelectricity in a Multiferroic Metal-Organic Framework. Angew. Chem., Int. Ed. 2011, 50, 5847–5850. 10.1002/anie.201101405. 21618371

[ref18] Zhou C.; Lin H.; He Q.; Xu L.; Worku M.; Chaaban M.; Lee S.; Shi X.; Du M.-H.; Ma B. Low Dimensional Metal Halide Perovskites and Hybrids. Mater. Sci. Eng., R 2019, 137, 38–65. 10.1016/j.mser.2018.12.001.

[ref19] Saidaminov M. I.; Mohammed O. F.; Bakr O. M. Low-Dimensional-Networked Metal Halide Perovskites: The Next Big Thing. ACS Energy Lett. 2017, 2, 889–896. 10.1021/acsenergylett.6b00705.

[ref20] Lin H.; Zhou C.; Tian Y.; Siegrist T.; Ma B. Low-Dimensional Organometal Halide Perovskites. ACS Energy Lett. 2018, 3, 54–62. 10.1021/acsenergylett.7b00926.

[ref21] Burger S.; Ehrenreich M. G.; Kieslich G. Tolerance Factors of Hybrid Organic–Inorganic Perovskites: Recent Improvements and Current State of Research. J. Mater. Chem. A 2018, 6, 21785–21793. 10.1039/C8TA05794J.

[ref22] Mao L.; Stoumpos C. C.; Kanatzidis M. G. Two-Dimensional Hybrid Halide Perovskites: Principles and Promises. J. Am. Chem. Soc. 2019, 141, 1171–1190. 10.1021/jacs.8b10851. 30399319

[ref23] Sun S.; Lu M.; Gao X.; Shi Z.; Bai X.; Yu W. W.; Zhang Y. 0D Perovskites: Unique Properties, Synthesis, and Their Applications. Adv. Sci. 2021, 8, 2102689 10.1002/advs.202102689. PMC8693037 34693663

[ref24] Han Y.; Yue S.; Cui B. Low-Dimensional Metal Halide Perovskite Crystal Materials: Structure Strategies and Luminescence Applications. Adv. Sci. 2021, 8, 2004805 10.1002/advs.202004805. PMC8336498 34137519

[ref25] Wang G.; Mei S.; Liao J.; Wang W.; Tang Y.; Zhang Q.; Tang Z.; Wu B.; Xing G. Advances of Nonlinear Photonics in Low-Dimensional Halide Perovskites. Small 2021, 17, 2100809 10.1002/smll.202100809. 34121324

[ref26] Wang Y.; Song L.; Chen Y.; Huang W. Emerging New-Generation Photodetectors Based on Low-Dimensional Halide Perovskites. ACS Photonics 2020, 7, 10–28. 10.1021/acsphotonics.9b01233.

[ref27] Willett R. D.; Gómez-García C. J.; Twamley B. Long-Range Order in Layered Perovskite Salts – Structure and Magnetic Properties of [(CH_3_)_2_CHCH_2_NH_3_]_2_CuX_4_ (X = Cl, Br). Eur. J. Inorg. Chem. 2012, 2012, 3342–3348. 10.1002/ejic.201101200.

[ref28] Sekine T.; Okuno T.; Awaga K. Observation of Spontaneous Magnetization in the Layered Perovskite Ferromagnet, (*p* -Chloroanilinium)_2_CuBr_4_. Inorg. Chem. 1998, 37, 2129–2133. 10.1021/ic970793z. 11670365

[ref29] Willett R. D.; Gómez-García C. J.; Twamley B. Structure and Magnetic Properties of [(REDA)Cl]_2_CuCl_4_ Salts: A New Series of Ferromagnetic Layer Perovskites. Polyhedron 2005, 24, 2293–2298. 10.1016/j.poly.2005.03.077.

[ref30] Asaji T. Structural and Magnetic Phase Transitions in (Chloroanilinium)_2_CuX_4_ (X = Cl, Br). Appl. Magn. Reson. 2004, 27, 197–205. 10.1007/BF03166314.

[ref31] Willett R. D.; Gómez-García C. J.; Twamley B.; Gómez-Coca S.; Ruiz E. Exchange Coupling Mediated by N–H···Cl Hydrogen Bonds: Experimental and Theoretical Study of the Frustrated Magnetic System in Bis(*o* -Phenylenediamine)Nickel(II) Chloride. Inorg. Chem. 2012, 51, 5487–5493. 10.1021/ic3007124. 22512477

[ref32] Han C.; Bradford A. J.; McNulty J. A.; Zhang W.; Halasyamani P. S.; Slawin A. M. Z.; Morrison F. D.; Lee S. L.; Lightfoot P. Polarity and Ferromagnetism in Two-Dimensional Hybrid Copper Perovskites with Chlorinated Aromatic Spacers. Chem. Mater. 2022, 34, 2458–2467. 10.1021/acs.chemmater.2c00107. 35431437 PMC9008537

[ref33] Han C.; McNulty J. A.; Bradford A. J.; Slawin A. M. Z.; Morrison F. D.; Lee S. L.; Lightfoot P. Polar Ferromagnet Induced by Fluorine Positioning in Isomeric Layered Copper Halide Perovskites. Inorg. Chem. 2022, 61, 3230–3239. 10.1021/acs.inorgchem.1c03726. 35138839 PMC9007457

[ref34] Šenjug P.; Dragović J.; Kalanj M.; Torić F.; Rubčić M.; Pajić D. Magnetic Behaviour of (C_2_H_5_NH_3_)_2_CuCl_4_ Type Multiferroic. J. Magn. Magn. Mater. 2019, 479, 144–148. 10.1016/j.jmmm.2019.02.020.

[ref35] Ai Y.; Sun R.; Liao W.; Song X.; Tang Y.; Wang B.; Wang Z.; Gao S.; Xiong R. Unprecedented Ferroelectricity and Ferromagnetism in a Cr^2+^-Based Two-Dimensional Hybrid Perovskite. Angew. Chem., Int. Ed. 2022, 61, e202206034 10.1002/anie.202206034. 35604204

[ref36] Gao Y.; Gao M.; Lu Y. Two-Dimensional Multiferroics. Nanoscale 2021, 13, 19324–19340. 10.1039/D1NR06598J. 34816857

[ref37] Sun B.; Liu X.-F.; Li X.-Y.; Zhang Y.; Shao X.; Yang D.; Zhang H.-L. Two-Dimensional Perovskite Chiral Ferromagnets. Chem. Mater. 2020, 32, 8914–8920. 10.1021/acs.chemmater.0c02729.

[ref38] Marchenko E. I.; Fateev S. A.; Petrov A. A.; Korolev V. V.; Mitrofanov A.; Petrov A. V.; Goodilin E. A.; Tarasov A. B. Database of Two-Dimensional Hybrid Perovskite Materials: Open-Access Collection of Crystal Structures, Band Gaps, and Atomic Partial Charges Predicted by Machine Learning. Chem. Mater. 2020, 32, 7383–7388. 10.1021/acs.chemmater.0c02290.

[ref39] McNulty J. A.; Lightfoot P. Structural Chemistry of Layered Lead Halide Perovskites Containing Single Octahedral Layers. IUCr J. 2021, 8, 485–513. 10.1107/S2052252521005418. PMC8256700 34258000

[ref40] Lyu R.; Moore C. E.; Liu T.; Yu Y.; Wu Y. Predictive Design Model for Low-Dimensional Organic–Inorganic Halide Perovskites Assisted by Machine Learning. J. Am. Chem. Soc. 2021, 143, 12766–12776. 10.1021/jacs.1c05441. 34357756

[ref41] CrysAlisPro, Agilent Technologies, Version 171.42.49.

[ref42] Sheldrick G. M. *SHELXT* – Integrated Space-Group and Crystal-Structure Determination. Acta Crystallogr., Sect. A: Found. Adv. 2015, 71, 3–8. 10.1107/S2053273314026370. 25537383 PMC4283466

[ref43] Sheldrick G. M. Crystal Structure Refinement with *SHELXL*. Acta Crystallogr., Sect. C: Struct. Chem. 2015, 71, 3–8. 10.1107/S2053229614024218. 25567568 PMC4294323

[ref44] Dolomanov O. v.; Bourhis L. J.; Gildea R. J.; Howard J. A. K.; Puschmann H. *OLEX2*: A Complete Structure Solution, Refinement and Analysis Program. J. Appl. Crystallogr. 2009, 42, 339–341. 10.1107/S0021889808042726.

[ref45] Momma K.; Izumi F. *VESTA3* for Three-Dimensional Visualization of Crystal, Volumetric and Morphology Data. J. Appl. Crystallogr. 2011, 44, 1272–1276. 10.1107/S0021889811038970.

[ref46] Altomare A.; Cuocci C.; Giacovazzo C.; Moliterni A.; Rizzi R.; Corriero N.; Falcicchio A. *EXPO2013*: A Kit of Tools for Phasing Crystal Structures from Powder Data. J. Appl. Crystallogr. 2013, 46, 1231–1235. 10.1107/S0021889813013113.

[ref47] Evans J. S. O. Advanced Input Files & Parametric Quantitative Analysis Using Topas. Mater. Sci. Forum 2010, 651, 1–9. 10.4028/www.scientific.net/MSF.651.1.

[ref48] Cheary R. W.; Coelho A. A Fundamental Parameters Approach to X-Ray Line-Profile Fitting. J. Appl. Crystallogr. 1992, 25, 109–121. 10.1107/S0021889891010804.

[ref49] Smidstrup S.; Markussen T.; Vancraeyveld P.; Wellendorff J.; Schneider J.; Gunst T.; Verstichel B.; Stradi D.; Khomyakov P. A.; Vej-Hansen U. G.; Lee M.-E.; Chill S. T.; Rasmussen F.; Penazzi G.; Corsetti F.; Ojanperä A.; Jensen K.; Palsgaard M. L. N.; Martinez U.; Blom A.; Brandbyge M.; Stokbro K. QuantumATK: An Integrated Platform of Electronic and Atomic-Scale Modelling Tools. J. Phys.: Condens. Matter 2020, 32, 015901 10.1088/1361-648X/ab4007. 31470430

[ref50] Fischer J. E.; Bendele G.; Dinnebier R.; Stephens P. W.; Lin C. L.; Bykovetz N.; Zhu Q. Structural Analysis of Fullerene and Fulleride Solids from Synchrotron X-Ray Powder Diffraction. J. Phys. Chem. Solids 1995, 56, 1445–1457. 10.1016/0022-3697(95)00082-8.

[ref51] Stephens P. W. Phenomenological Model of Anisotropic Peak Broadening in Powder Diffraction. J. Appl. Crystallogr. 1999, 32, 281–289. 10.1107/S0021889898006001.

[ref52] Jayatilaka D.; Grimwood D. J. Tonto: A Fortran Based Object-Oriented System for Quantum Chemistry and Crystallography; 2003; pp 142–151.

[ref53] Lufaso M. W.; Woodward P. M. Jahn–Teller Distortions, Cation Ordering and Octahedral Tilting in Perovskites. Acta Crystallogr., Sect. B: Struct. Sci. 2004, 60, 10–20. 10.1107/S0108768103026661. 14734840

[ref54] Zanchini C.; Willett R. D. Crystal Structure, Magnetism, and Electronic and EPR Spectroscopies of Bis(2-Aminopyrimidinium) Tetrachlorocuprate(II): A Square-Planar CuCl42- Anion with Semicoordinated Cationic Ligands. Inorg. Chem. 1990, 29, 3027–3030. 10.1021/ic00341a034.

[ref55] Kersen Ü.; Wojtczak A.; Bienko A.; Jezierska J. The Effects of Protonated Heterocyclic Cations on the Structural and Magnetic Properties of Tetrachlorocuprate(II) Anions; X-Ray, Magnetochemical and EPR Studies. New J. Chem. 2018, 42, 15705–15713. 10.1039/C8NJ03155J.

[ref56] Turnbull M. M.; Galeriu C.; Giantsidis J.; Landee C. P. Synthesis, Structure and Magnetic Susceptibility of Two 5-Nitro-2-Aminopyridinium Cuprates: (5-NAP)_2_CuCl_4_ and the Quantum Magnetic Ladder (5-NAP)_2_CuBr_4_·H_2_O. Mol. Cryst. Liq. Cryst. 2002, 376, 469–476. 10.1080/10587250210779.

[ref57] Krasinski C. A.; Solomon B. L.; Awwadi F. F.; Landee C. P.; Turnbull M. M.; Wikaira J. L. Copper(II) Halide Salts and Complexes of 4-Amino-2-Fluoropyridine: Synthesis, Structure and Magnetic Properties. J. Coord. Chem. 2017, 70, 914–935. 10.1080/00958972.2016.1278213.

[ref58] **(*p*-A)_2_CuCl_4_** displays two types of octahedral tilt modes, X_2_^+^ and X_3_^+^, without layer shift, resulting in the tilt system *a*^–^*a*^–^*c*/–(*a*^–^*a*^–^)*c* according to the Glazer notation. The situation for **(*m*-A)_2_CuCl_4_** is far more complex, due to stacking faults present in the structure, and the assignment of the corresponding modes is not that straightforward. For more details regarding distortion modes analysis see ref (39).

[ref59] Polyakov A. O.; Arkenbout A. H.; Baas J.; Blake G. R.; Meetsma A.; Caretta A.; van Loosdrecht P. H. M.; Palstra T. T. M. Coexisting Ferromagnetic and Ferroelectric Order in a CuCl_4_ -Based Organic–Inorganic Hybrid. Chem. Mater. 2012, 24, 133–139. 10.1021/cm2023696.

[ref60] de Jongh L. J. Observation of Lattice- and Spin-Dimensionality Crossovers in the Susceptibility of Quasi 2-Dimensional Heisenberg Ferromagnets. Phys. B+C 1976, 82, 247–261. 10.1016/0378-4363(76)90187-X.

[ref61] Bonner J. C.; Fisher M. E. Linear Magnetic Chains with Anisotropic Coupling. Phys. Rev. 1964, 135, A640–A658. 10.1103/PhysRev.135.A640.

