# Supplementary material for: Structure-Related Evolution of Magnetic Order in Anisidinium Tetrachlorocuprates(II)
Source: Cryst Growth Des. 2023 May 9;23(6):4262–72. doi: 10.1021/acs.cgd.3c00066 (PMC10251751; doi:10.1021/acs.cgd.3c00066)
Supplement: Supplementary file 1 — cg3c00066_si_001.pdf [file cg3c00066_si_001.pdf]

Structure-related evolution of magnetic order in anisidinium tetrachlorocuprates(II)

Edi Topić,[1] Pavla Šenjug,[2] Dario Barišić,[2] Ivor Lončarić,[3] Damir Pajić[2] and Mirta Rubčić*[1]*

1 – Department of Chemistry, Faculty of Science, University of Zagreb, Horvatovac 102a, 10000 Zagreb, Croatia
2 – Department of Physics, Faculty of Science, University of Zagreb, Bijenička cesta 32, 10000 Zagreb, Croatia
3 – Ruđer Bošković Institute, Bijenička cesta 54, 10000 Zagreb, Croatia

Supporting information

Contents

Experimental section – X-ray diffraction **(*m*-A*)$_2$CuCl$_4$** 2
Experimental section - Thermogravimetric analysis and FTIR spectroscopy 4
Experimental section – X-ray diffraction 6
Single-crystal X-ray diffraction 6
Powder X-ray diffraction 8
Powder X-ray diffraction – *TOPAS* input files 11
Results and discussion 17
Dimensionality calculation 18
Bounding box calculations 23
References 25

Experimental section – X-ray diffraction **(*m*-A*)$_2$CuCl$_4$**

The partially chlorinated compound **(*m*-A*)$_2$CuCl$_4$** was typically obtained in mixtures with the **(*m*-A)$_2$CuCl$_4$**, which made the characterization of bulk material demanding. For the purpose of the single-crystal X-ray diffraction, several crystals of the pure material **(*m*-A*)$_2$CuCl$_4$** were separated out from the mixture, visually inspected and analyzed. The refinement of the **(*m*-A*)$_2$CuCl$_4$** crystal structure unveiled the case of tetrachlorocuprate(II) with the layered hybrid structure, where the *m*-anisidinium cation was found to be partially chlorinated at position 4 on the aromatic ring, based on the refinement of the occupancy for the chlorine atom (refinement converged to a value of » 0.4). Another way of interpreting this result is that the organic layer in **(*m*-A*)$_2$CuCl$_4$** is composed of two types of cations, chlorinated and non-chlorinated one in the approximate ratio 0.4 : 0.6.

Table S1. Experimental and crystallographic data for partially chlorinated **(*m*-A*)$_2$CuCl$_4$**

Compound	**(*m*-A*)$_2$CuCl$_4$**
Empirical formula	$C_{14}H_{18}N_2O_2CuCl_{4.79}$
M_r	479.74
T/K	293(2)
Crystal system	monoclinic, green prisms
Space group	C2/*c*
a/Å	34.121(3)
b/Å	8.1424(9)
c/Å	7.0199(7)
α/°	90
β/°	95.055(8)
γ/°	90
V/Å^3	1942.8(3)
Z	4
ρ_{calc}/g cm^{-3}	1.640
μ/mm^{-1}	1.792
F(000)	970.0
Crystal size/mm^3	0.306 × 0.15 × 0.033
Radiation	MoKα (λ = 0.71073)
2Θ range/°	8.262 to 51.988
Index ranges	$-41 \leq h \leq 42$,<br>$-10 \leq k \leq 10$,<br>$-8 \leq l \leq 8$
Reflections collected	8936

Independent reflections	1901 [R_{int} = 0.1673, R_{sigma} = 0.1613]
Data/restraints/ parameters	1901/0/118
Goodness–of–fit on F^2, S^b	0.930
Final R and wR^c values [$I \geq 2\sigma(I)$]	R_1 = 0.0628, wR_2 = 0.0879
Final R and wR^c values [all data]	R_1 = 0.1735, wR_2 = 0.1141
Largest diff. peak/hole / e Å^{-3}	0.57/–0.38

$^a w = 1/[\sigma^2(F_o^2) + (g_1P)^2 + g_2P]$ where $P = (F_o^2 + 2F_c^2)/3$

$^b S = \{\Sigma[w(F_o^2 - F_c^2)^2]/(N_r - N_p)\}^{1/2}$ where N_r = number of independent reflections, N_p = number of refined parameters.

$^c R = \Sigma||F_o| - |F_c||/\Sigma|F_o|$; $wR = \{\Sigma[w(F_o^2 - F_c^2)^2]/\Sigma[w(F_o^2)^2]\}^{1/2}$

Experimental section - Thermogravimetric analysis and FTIR spectroscopy

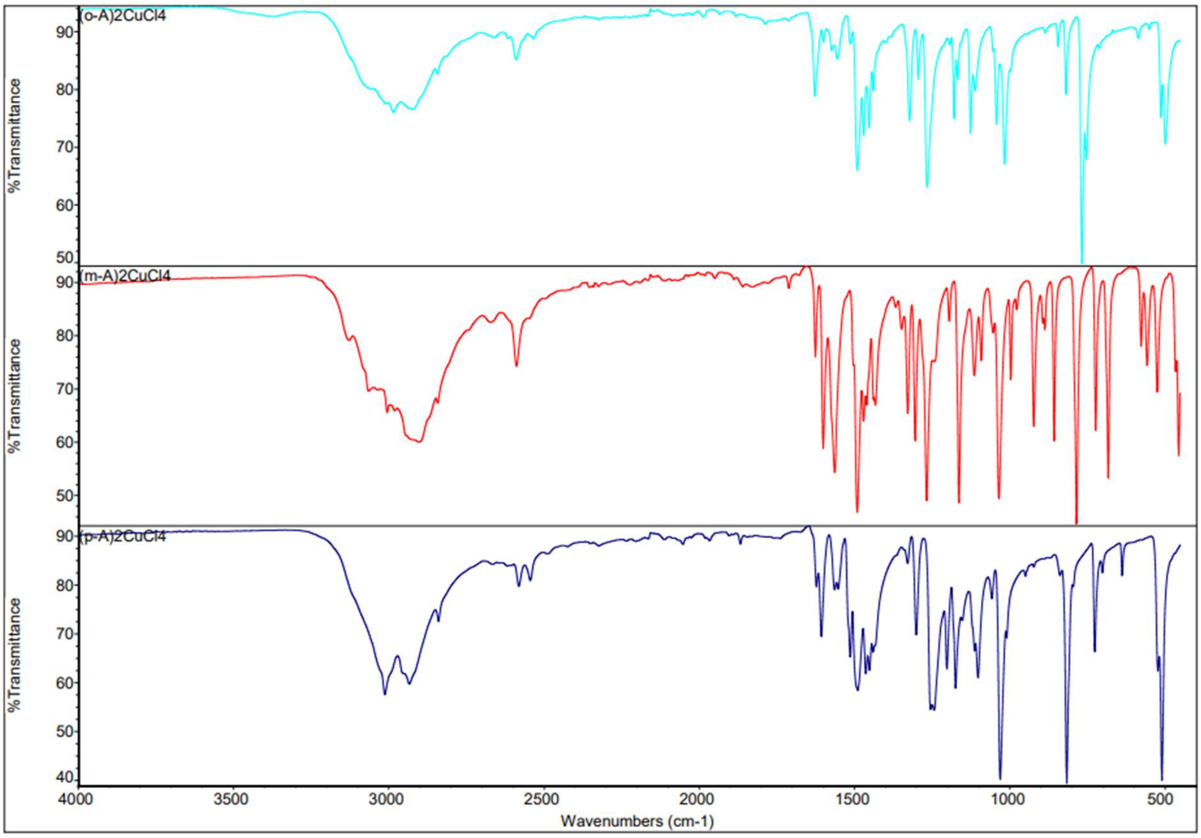

Figure S1. FTIR spectra of **(*o*-A)$_2$CuCl$_4$** (top), **(*m*-A)$_2$CuCl$_4$** (middle) and **(*p*-A)$_2$CuCl$_4$** (bottom).

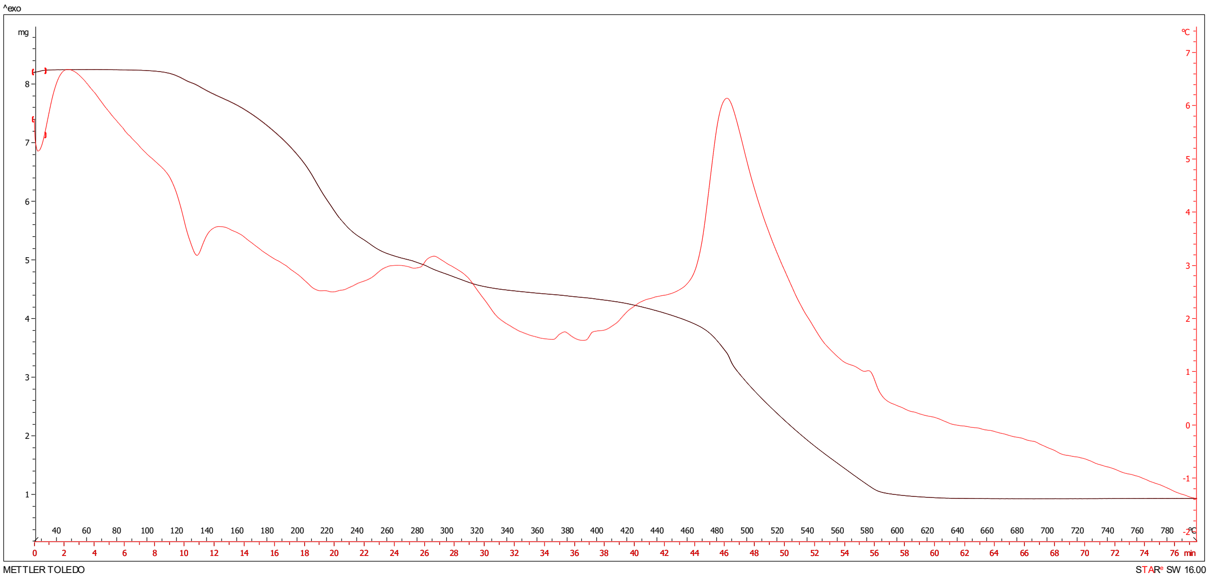

Figure S2. TGA (black) and DTA (red) curve of **(*o*-A)$_2$CuCl$_4$**. The compound is stable up to *cca.* 120 °C, with complex decomposition occuring afterwards.

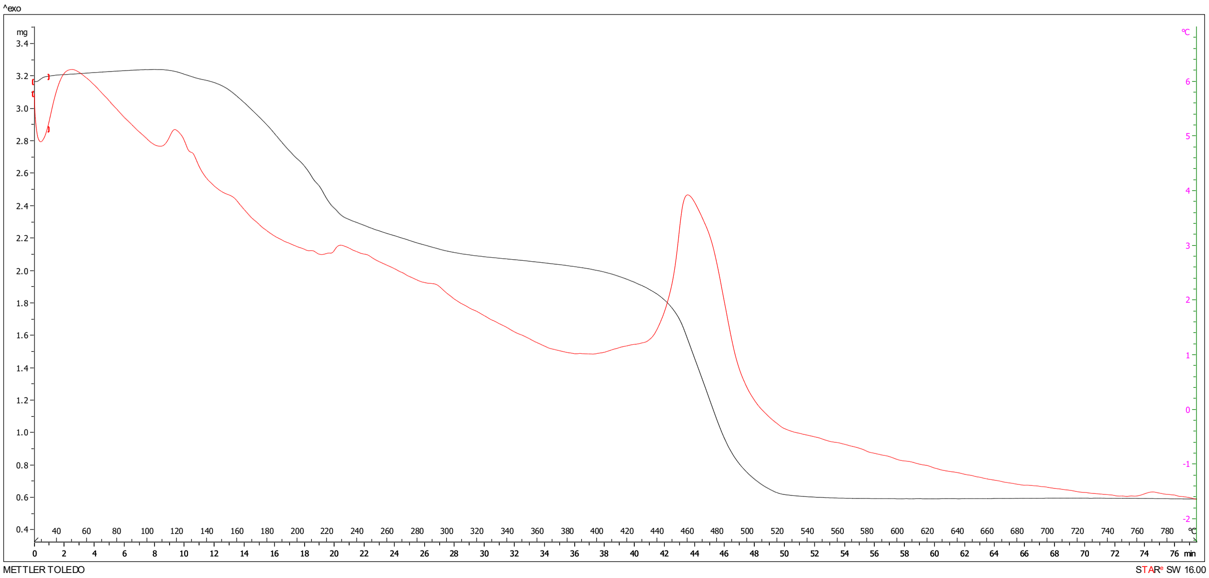

Figure S3. TGA (black) and DTA (red) curve of **(*m*-A)$_2$CuCl$_4$**. The compound is stable up to *cca.* 110 °C, with complex decomposition occuring afterwards.

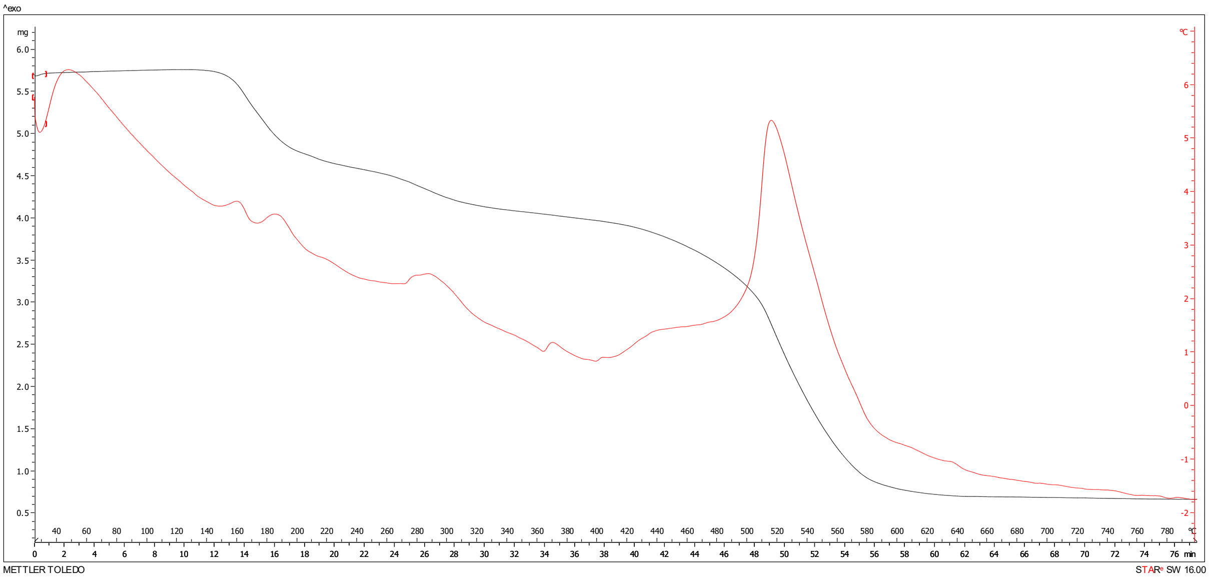

Figure S4. TGA (black) and DTA (red) curve of **(*p*-A)$_2$CuCl$_4$**. The compound is stable up to *cca.* 140 °C, with complex decomposition occuring afterwards.

Experimental section – X-ray diffraction

Single-crystal X-ray diffraction

Table S2. Experimental and crystallographic data for prepared compounds

Compound	**(*o*-A)$_2$CuCl$_4$**	**(*m*-A)$_2$CuCl$_4$***	**(*p*-A)$_2$CuCl$_4$**
Empirical formula	$C_{14}H_{20}N_2O_2CuCl_4$		
M_r	453.66	453.66	453.66
T/K	298(2)	298(2)	298(2)
Crystal system	monoclinic, dark-green prism	monoclinic, light green powder	orthorhombic, gold plates
Space group	$P2_1/n$	$P2_1/c$	$Pbca$
a/Å	10.8129(4)	8.10490(13)	7.4006(2)
b/Å	7.6060(2)	7.33725(19)	7.1575(2)
c/Å	12.1023(4)	31.4334(3)	36.1667(12)
α/°	90	90	90
β/°	111.605(4)	96.7199(17)	90
γ/°	90	90	90
V/Å^3	925.40(6)	1856.43(6)	1915.74(10)
Z	2	4	4
ρ_{calc}/g cm^{-3}	1.628	1.623	1.573
μ/mm^{-1}	1.766	-	1.706
F(000)	462	924	924
Crystal size/mm^3	0.705 × 0.287 × 0.13	powder sample	0.384 × 0.292 × 0.038
Radiation	Mo Kα (λ = 0.71073 Å)	Cu Kα1 (1.540596 Å)	Mo Kα (λ = 0.71073 Å)
2Θ range/°	8.66 to 65.724	4.00 to 68.00	8.236 to 65.214
Index ranges	$-14 \le h \le 15$, $-11 \le k \le 11$, $-17 \le l \le 18$	-	$-11 \le h \le 11$, $-10 \le k \le 10$, $-45 \le l \le 53$
Reflections collected	10764	-	22261
Independent reflections	3132 [R_{int} = 0.0231, R_{sigma} = 0.0261]	-	3294 [R_{int} = 0.0529, R_{sigma} = 0.0382]
Data/restraints/ parameters	3132/3/119	9142/-/56	3294/3/116
Goodness–of–fit on F^2, S^b	1.029	4.59 (uncorrected)	1.151
Final R and wR^c values [$I \ge 2\sigma(I)$]	R_1 = 0.0327, wR_2 = 0.0791	-	R_1 = 0.0506, wR_2 = 0.1055
Final R and wR^c values [all data]	R_1 = 0.0485, wR_2 = 0.0886	R_p = 0.074, R_{wp} = 0.110	R_1 = 0.0814, wR_2 = 0.1175

Largest diff. peak/hole / e Å^{-3}	0.47/–0.38	-	0.67/–0.45

* solved from powder diffraction data – for comparison only

$^{a}w = 1/[\sigma^2(F_o^2) + (g_1P)^2 + g_2P]$ where $P = (F_o^2 + 2F_c^2)/3$

$^{b}S = \{\Sigma[w(F_o^2 - F_c^2)^2]/(N_r - N_p)\}^{1/2}$ where N_r = number of independent reflections, N_p = number of refined parameters.

$^{c}R = \Sigma||F_o| - |F_c||/\Sigma|F_o|$; $wR = \{\Sigma[w(F_o^2 - F_c^2)^2]/\Sigma[w(F_o^2)^2]\}^{1/2}$

Powder X-ray diffraction

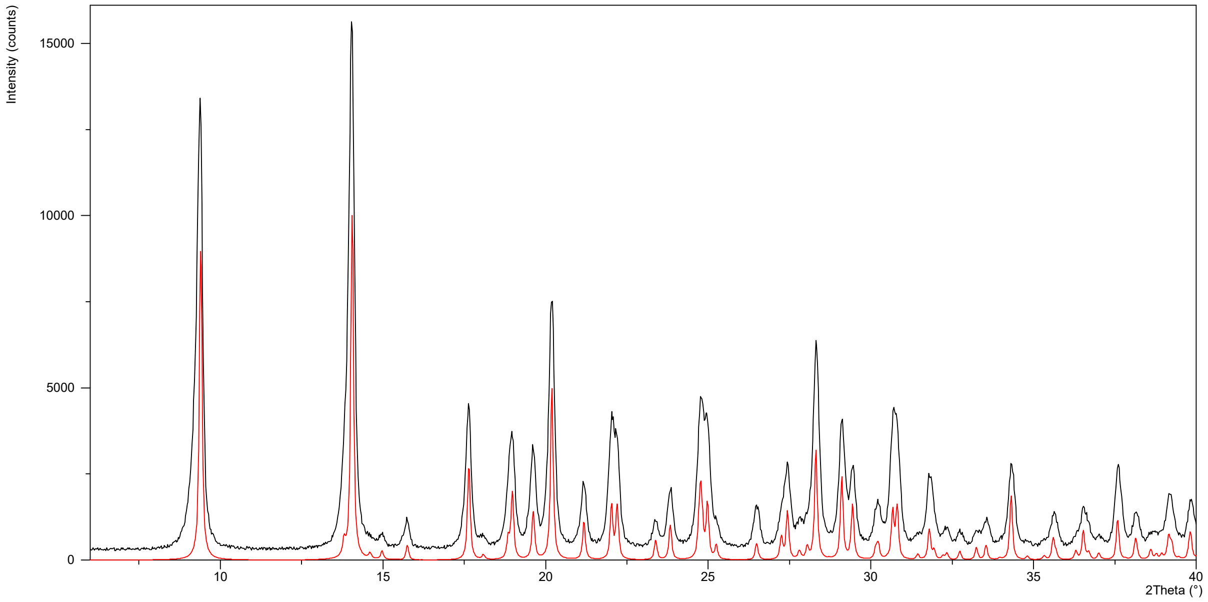

(a)

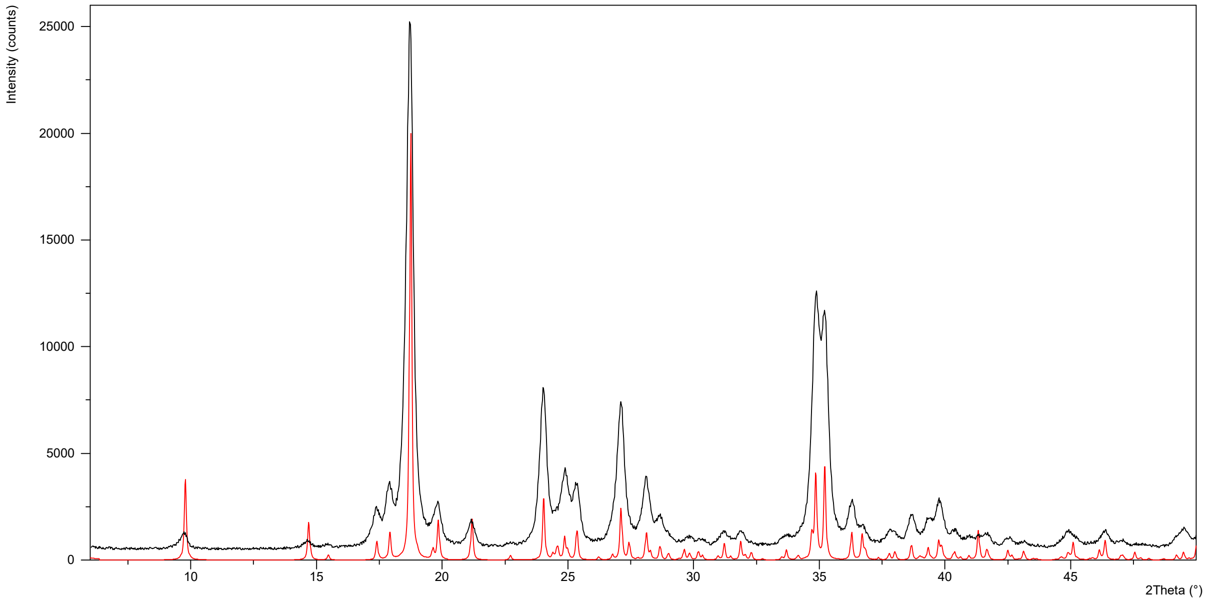

(b)

Figure S5. Measured (black) and calculated (red) PXRD data for (a) **(*o*-A)$_2$CuCl$_4$**, (b) **(*p*-A)$_2$CuCl$_4$** . The measured pattern shows good agreement with the calculated one, indicating the phase purity of the bulk sample.

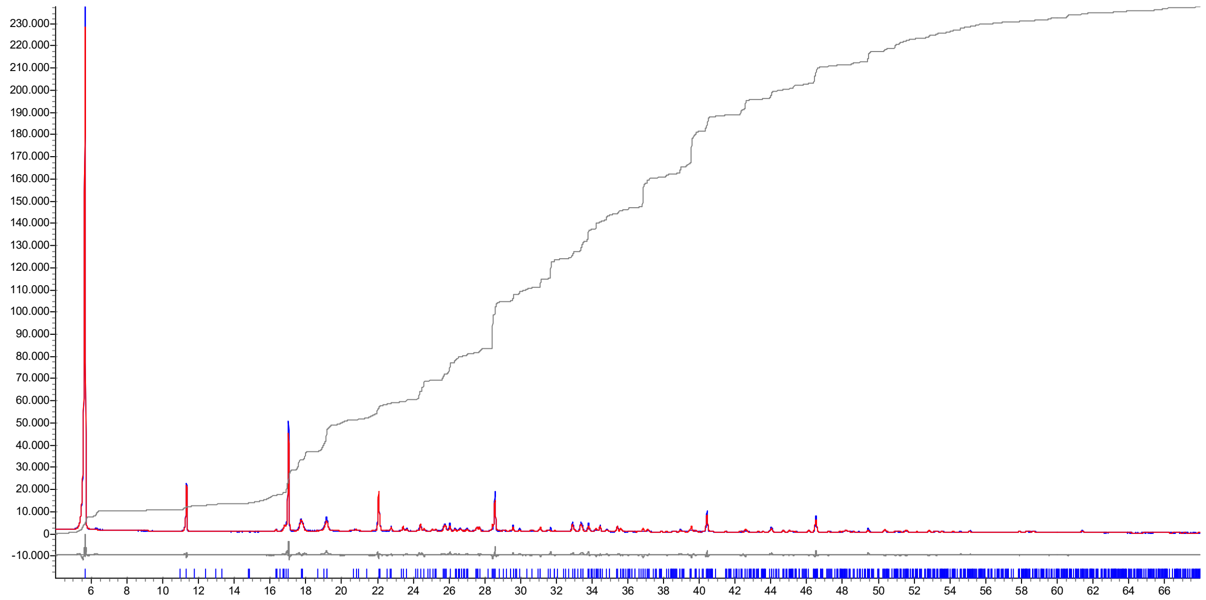

Figure S6. Results of Rietveld refinement of **(*m*-A)$_2$CuCl$_4$** powder data using Stephens *hkl*-dependent peak width approach. Blue line represents the measured intensities (Y_{obs}), red line represents calculated intensities (Y_{calc}), grey line below the pattern represents differences (Y_{obs}–Y_{calc}) and the grey line above the pattern represents cumulative χ^2.

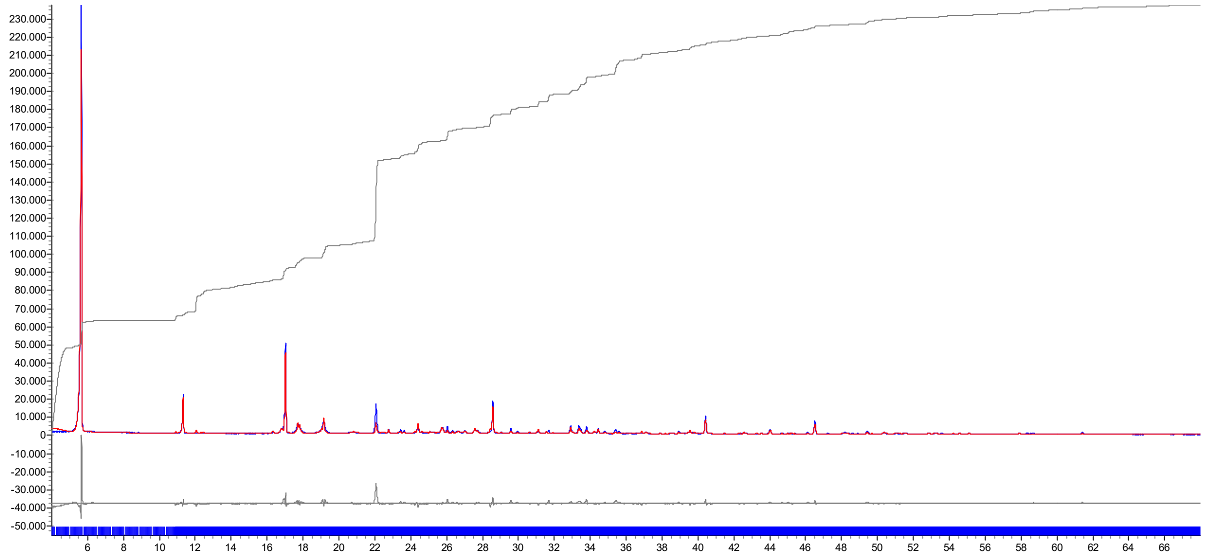

Figure S7. Results of Rietveld refinement of **(*m*-A)$_2$CuCl$_4$** powder data using stacking faults (N_{cells} = 50) approach. Blue line represents the measured intensities (Y_{obs}), red line represents calculated intensities (Y_{calc}), grey line below the pattern represents differences (Y_{obs}–Y_{calc}) and the grey line above the pattern represents cumulative χ^2.

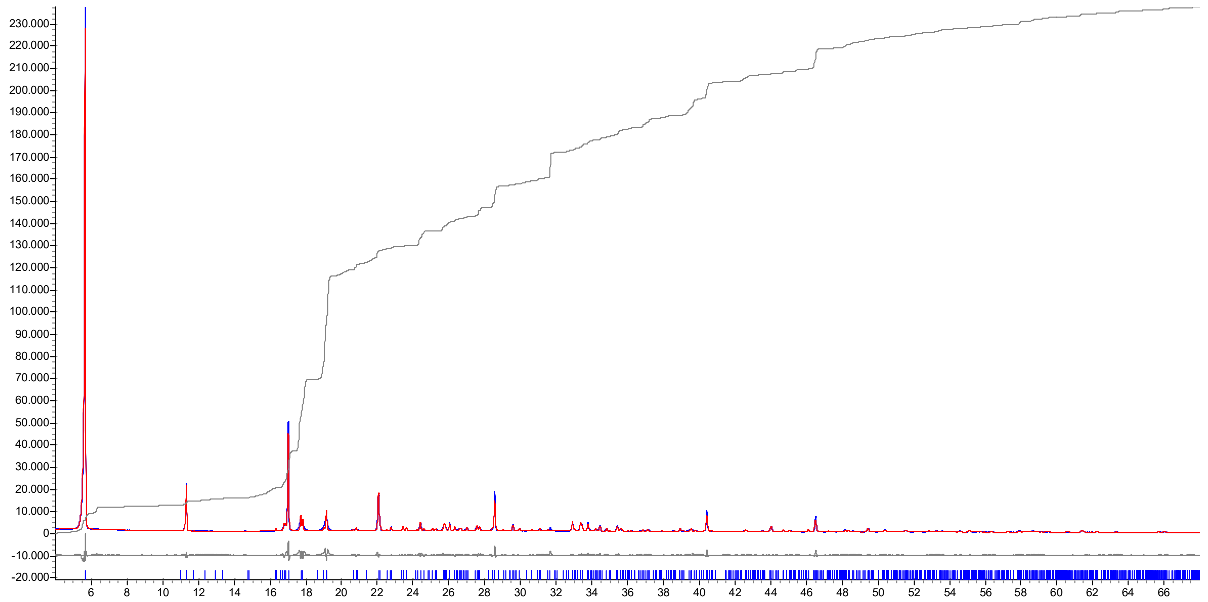

Figure S8. Results of Rietveld refinement of **(*m*-A)$_2$CuCl$_4$** powder data using spherical harmonics approach. Blue line represents the measured intensities (Y_{obs}), red line represents calculated intensities (Y_{calc}), grey line below the pattern represents differences (Y_{obs}–Y_{calc}) and the grey line above the pattern represents cumulative χ^2.

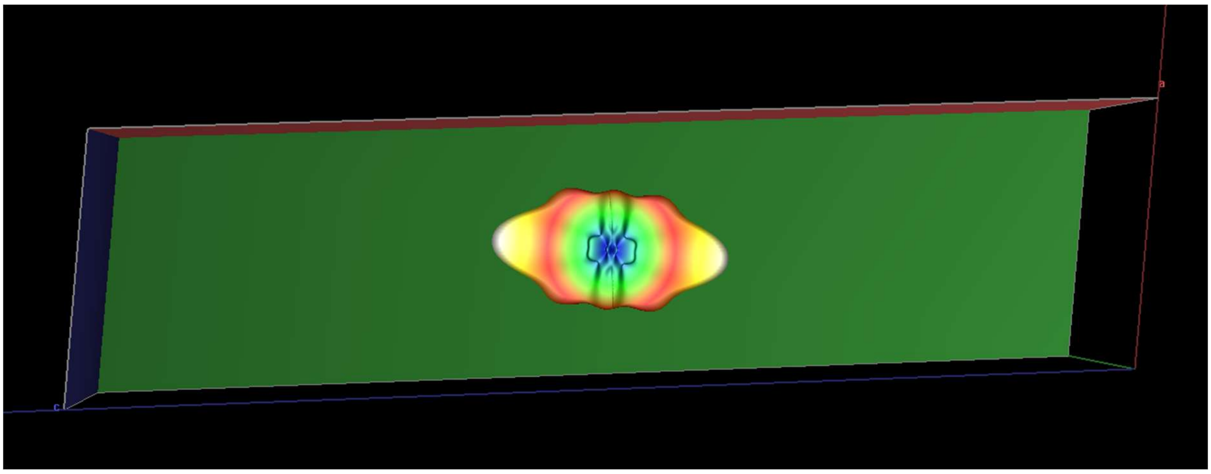

Figure S9. Sum of spherical harmonics obtained by Rietveld refinement represented in *ac* plane.

Table S3. Powder data refinement statistics for different anisotropic peak width approaches

Approach	Spherical harmonics	Stephens	Stacking faults<br>Average fractional displacement:<br>*a* 0.034, *b* 0.063, *c* 0	Preferred orientation only
R_{wp}/%	11.07	11.34	13.72	13.13
GoF/%	4.59	4.70	5.72	5.44

Powder X-ray diffraction – *TOPAS* input files

Final refinement stage (Spherical harmonics approach)

```
iters 20000
xdd maniscucl4.xye
'continue_after_convergence
do_errors
         r_wp  11.0733771 r_exp  2.41077666 r_p  7.43056281 r_wp_dash  19.1736521 r_p_dash  16.7240462 r_exp_dash
4.17428147 weighted_Durbin_Watson  0.101578122 gof  4.59328203
    lam
         ymin_on_ymax 0.0001
      la 1 lo 1.540596 lh 0.5
         bkg @  1024.03557`_2.77149491 -694.968676`_4.38430834  130.4212`_4.27313082 -58.3739852`_4.03745113
129.641286`_3.86431246 -148.359465`_3.76370342  46.3104605`_3.77075888  72.9945541`_3.75071398 -75.4270563`_3.84451563
52.6520624`_3.80909181 -35.3082453`_3.81109293  20.9203027`_3.69441655  0.857865301`_3.65704717 -2.29258716`_3.46443261 -
0.881058117`_3.29729544  36.3410296`_3.07815299 -28.5929938`_3.00587627  25.8697397`_2.73265111  11.1830623`_2.54179303
         Radius(240)
         Full_Axial_Model(12, 15, 12, 2.3,  @  2.96990`_0.01333)
         Zero_Error(@, 0.00587`_0.00014)
         LP_Factor(26.4)
         str
                  scale @  0.000198582183`_0.0002586
                  space_group "P21/c"
                  a  @  8.104902`_0.000126 min 8 max 8.2
                  b  @  7.337254`_0.000187 min 7.2 max 7.4
                  c  @  31.433402`_0.000342
                  al 90
                  be @  96.71972`_0.00171 min 96.6 max 96.8
                  ga 90
                  volume  1856.432`_0.059
                  prm beq1  1.50776`_0.16531
                  prm beq2  1.16829`_0.15135
                  prm beq3  0.50604`_0.17063
                  site Cu1    x 0.7520       y 0.8482       z 0.74972      occ Cu   1 beq =beq1;
                  site Cl1    x 0.9390       y 1.0900       z 0.76261      occ Cl   1 beq =beq2;
                  site Cl2    x 0.7254       y 0.9111       z 0.67932      occ Cl   1 beq =beq2;
                  site Cl3    x 0.5649       y 0.6063       z 0.73684      occ Cl   1 beq =beq2;
                  site Cl4    x 0.7785       y 0.7852       z 0.82013      occ Cl   1 beq =beq2;
                  site O1     x 0.391        y 0.21         z 0.0478       occ O    1 beq =beq3;
                  site N1     x 0.765        y 0.120        z 0.1748       occ N    1 beq =beq3;
                  site C1     x 0.7355       y 0.120        z 0.1323       occ C    1 beq =beq3;
                  site C2     x 0.578        y 0.164        z 0.1122       occ C    1 beq =beq3;
                  site C3     x 0.547        y 0.164        z 0.0676       occ C    1 beq =beq3;
                  site C4     x 0.6742       y 0.120        z 0.0430       occ C    1 beq =beq3;
                  site C5     x 0.832        y 0.076        z 0.0631       occ C    1 beq =beq3;
                  site C6     x 0.863        y 0.076        z 0.1077       occ C    1 beq =beq3;
                  site C7     x 0.272        y 0.25         z 0.0698       occ C    1 beq =beq3;
                  site H1     x 0.83         y 0.036        z 0.1877       occ H    1 beq =beq3*1.5;
                  site H2     x 0.81         y 0.212        z 0.1879       occ H    1 beq =beq3*1.5;
                  site H3     x 0.683        y 0.11         z 0.1900       occ H    1 beq =beq3*1.5;
                  site H4     x 0.24         y 0.39         z 0.075        occ H    1 beq =beq3*1.5;
                  site H5     x 0.144        y 0.20         z 0.059        occ H    1 beq =beq3*1.5;
                  site H6     x 0.277        y 0.20         z 0.1037       occ H    1 beq =beq3*1.5;
                  site H7     x 0.478        y 0.198        z 0.1314       occ H    1 beq =beq3*1.5;
                  site H8     x 0.6502       y 0.120        z 0.0081       occ H    1 beq =beq3*1.5;
                  site H9     x 0.931        y 0.042        z 0.0439       occ H    1 beq =beq3*1.5;
                  site H10    x 0.986        y 0.042        z 0.1234       occ H    1 beq =beq3*1.5;
                  site O1a    x -0.101       y 0.90         z 0.5443       occ O    1 beq =beq3;
                  site N1a    x 0.254        y 0.822        z 0.6821       occ N    1 beq =beq3;
                  site C1a    x 0.2306       y 0.822        z 0.6331       occ C    1 beq =beq3;
                  site C2a    x 0.075        y 0.860        z 0.6110       occ C    1 beq =beq3;
                  site C3a    x 0.053        y 0.860        z 0.5663       occ C    1 beq =beq3;
                  site C4a    x 0.1876       y 0.822        z 0.5436       occ C    1 beq =beq3;
                  site C5a    x 0.343        y 0.783        z 0.5657       occ C    1 beq =beq3;
                  site C6a    x 0.365        y 0.783        z 0.6105       occ C    1 beq =beq3;
                  site C7a    x -0.238       y 0.93         z 0.5660       occ C    1 beq =beq3;
                  site H1a    x 0.191        y 0.888        z 0.6960       occ H    1 beq =beq3*1.5;
                  site H2a    x 0.24         y 0.722        z 0.6959       occ H    1 beq =beq3*1.5;
                  site H3a    x 0.347        y 0.86         z 0.6957       occ H    1 beq =beq3*1.5;
                  site H4a    x -0.343       y 0.84         z 0.564        occ H    1 beq =beq3*1.5;
                  site H5a    x -0.221       y 0.95         z 0.6014       occ H    1 beq =beq3*1.5;
                  site H6a    x -0.31        y 1.06         z 0.559        occ H    1 beq =beq3*1.5;
                  site H7a    x -0.030       y 0.89         z 0.6286       occ H    1 beq =beq3*1.5;
                  site H8a    x 0.171        y 0.822        z 0.5087       occ H    1 beq =beq3*1.5;
                  site H9a    x 0.448        y 0.75         z 0.5481       occ H    1 beq =beq3*1.5;
                  site H10a   x 0.486        y 0.75         z 0.6277       occ H    1 beq =beq3*1.5;


                  CS_G(@, 237.65543`_1.50962)
                  Strain_L(@, 0.06248`_0.00304)
                  Strain_G(@, 0.17820`_0.00321)


                  Out_CIF_STR("m-anis2cucl4.cif")

                  PO_Spherical_Harmonics(sh, 8 load sh_Cij_prm {
                           y00   !sh_c00  1.00000
                           y20   sh_c20   0.21763`_2.58501
                           y22p  sh_c22p -0.65758`_2.96443
                           y22m  sh_c22m -0.73399`_0.67320
                           y40   sh_c40   0.03561`_1.30739
                           y42p  sh_c42p -0.40977`_2.11966
                           y42m  sh_c42m -0.28600`_0.52318
                           y44p  sh_c44p -0.04536`_0.68811
                           y44m  sh_c44m  0.36784`_3.38969
                           y60   sh_c60  -0.20656`_0.30178
```

```
                    y62p  sh_c62p -0.18300`_0.32373
                    y62m  sh_c62m -0.07689`_0.06390
                    y64p  sh_c64p  0.43924`_0.69368
                    y64m  sh_c64m -0.06223`_0.64233
                    y66p  sh_c66p -0.28193`_1.72602
                    y66m  sh_c66m -0.63235`_1.28576
                    y80   sh_c80   0.28589`_0.36985
                    y82p  sh_c82p  0.23317`_0.30106
                    y82m  sh_c82m  0.04642`_0.05586
                    y84p  sh_c84p  0.17984`_0.21893
                    y84m  sh_c84m -0.10678`_0.13841
                    y86p  sh_c86p -0.09193`_0.08019
                    y86m  sh_c86m  0.14358`_0.22640
                    y88p  sh_c88p  0.46581`_0.77055
                    y88m  sh_c88m  0.08579`_0.20993
                    } )
          normals_plot = sh;
          normals_plot_min_d .25
```

Final refinement stage (Stephens *hkl*-dependent peak width approach)

```
iters 20000
xdd maniscucl4.xye
'continue_after_convergence
do_errors
          r_wp  11.8969245 r_exp  2.4160774 r_p  8.09936892 r_wp_dash  20.6045191 r_p_dash  18.2420487 r_exp_dash  4.18445231
weighted_Durbin_Watson  0.0805357639 gof  4.92406594
   lam
          ymin_on_ymax 0.0001
      la 1 lo 1.540596 lh 0.5
          bkg   1024.52891_2.77979026 -694.276759_4.39233654  130.271762_4.27767137 -58.6976779_4.04124337
129.469875_3.86828411 -148.324962_3.76727146  46.2064912_3.77468557  72.8402637_3.75483386 -75.582916_3.84842719
52.5406367_3.81248689 -35.2429827_3.81408957  20.9836266_3.69739579  0.616606105_3.66159744 -2.61093015_3.46877591 -
1.04597157_3.30066941  36.4111447_3.08096665 -28.5670008_3.00912253  25.905611_2.73546326  11.183572_2.54459659
          Radius(240)
          Full_Axial_Model(12, 15, 12, 2.3,    2.96984_0.01332)
          Zero_Error(, 0.00589_0.00014)
          LP_Factor(26.4)
          str
                    scale @  0.000177932133`_6.783e-007
                    space_group "P21/c"
                    a    8.104918_0.000125 min 8 max 8.2
                    b    7.337263_0.000185 min 7.2 max 7.4
                    c    31.433453_0.000338
                    al 90
                    be   96.71971_0.00167 min 96.6 max 96.8
                    ga 90
                    volume  1856.441
                    prm !beq1  1.49035_0.16250
                    prm !beq2  0.86661_0.14618
                    prm !beq3  0.43503_0.16833
                    site Cu1    x 0.7520       y 0.8482       z 0.74972      occ Cu   1 beq =beq1;
                    site Cl1    x 0.9390       y 1.0900       z 0.76261      occ Cl   1 beq =beq2;
                    site Cl2    x 0.7254       y 0.9111       z 0.67932      occ Cl   1 beq =beq2;
                    site Cl3    x 0.5649       y 0.6063       z 0.73684      occ Cl   1 beq =beq2;
                    site Cl4    x 0.7785       y 0.7852       z 0.82013      occ Cl   1 beq =beq2;
                    site O1     x 0.391        y 0.21         z 0.0478       occ O    1 beq =beq3;
                    site N1     x 0.765        y 0.120        z 0.1748       occ N    1 beq =beq3;
                    site C1     x 0.7355       y 0.120        z 0.1323       occ C    1 beq =beq3;
                    site C2     x 0.578        y 0.164        z 0.1122       occ C    1 beq =beq3;
                    site C3     x 0.547        y 0.164        z 0.0676       occ C    1 beq =beq3;
                    site C4     x 0.6742       y 0.120        z 0.0430       occ C    1 beq =beq3;
                    site C5     x 0.832        y 0.076        z 0.0631       occ C    1 beq =beq3;
                    site C6     x 0.863        y 0.076        z 0.1077       occ C    1 beq =beq3;
                    site C7     x 0.272        y 0.25         z 0.0698       occ C    1 beq =beq3;
                    site H1     x 0.83         y 0.036        z 0.1877       occ H    1 beq =beq3*1.5;
                    site H2     x 0.81         y 0.212        z 0.1879       occ H    1 beq =beq3*1.5;
                    site H3     x 0.683        y 0.11         z 0.1900       occ H    1 beq =beq3*1.5;
                    site H4     x 0.24         y 0.39         z 0.075        occ H    1 beq =beq3*1.5;
                    site H5     x 0.144        y 0.20         z 0.059        occ H    1 beq =beq3*1.5;
                    site H6     x 0.277        y 0.20         z 0.1037       occ H    1 beq =beq3*1.5;
                    site H7     x 0.478        y 0.198        z 0.1314       occ H    1 beq =beq3*1.5;
                    site H8     x 0.6502       y 0.120        z 0.0081       occ H    1 beq =beq3*1.5;
                    site H9     x 0.931        y 0.042        z 0.0439       occ H    1 beq =beq3*1.5;
                    site H10    x 0.986        y 0.042        z 0.1234       occ H    1 beq =beq3*1.5;
                    site O1a    x -0.101       y 0.90         z 0.5443       occ O    1 beq =beq3;
                    site N1a    x 0.254        y 0.822        z 0.6821       occ N    1 beq =beq3;
                    site C1a    x 0.2306       y 0.822        z 0.6331       occ C    1 beq =beq3;
                    site C2a    x 0.075        y 0.860        z 0.6110       occ C    1 beq =beq3;
                    site C3a    x 0.053        y 0.860        z 0.5663       occ C    1 beq =beq3;
                    site C4a    x 0.1876       y 0.822        z 0.5436       occ C    1 beq =beq3;
                    site C5a    x 0.343        y 0.783        z 0.5657       occ C    1 beq =beq3;
                    site C6a    x 0.365        y 0.783        z 0.6105       occ C    1 beq =beq3;
                    site C7a    x -0.238       y 0.93         z 0.5660       occ C    1 beq =beq3;
                    site H1a    x 0.191        y 0.888        z 0.6960       occ H    1 beq =beq3*1.5;
                    site H2a    x 0.24         y 0.722        z 0.6959       occ H    1 beq =beq3*1.5;
                    site H3a    x 0.347        y 0.86         z 0.6957       occ H    1 beq =beq3*1.5;
                    site H4a    x -0.343       y 0.84         z 0.564        occ H    1 beq =beq3*1.5;
                    site H5a    x -0.221       y 0.95         z 0.6014       occ H    1 beq =beq3*1.5;
                    site H6a    x -0.31        y 1.06         z 0.559        occ H    1 beq =beq3*1.5;
                    site H7a    x -0.030       y 0.89         z 0.6286       occ H    1 beq =beq3*1.5;
                    site H8a    x 0.171        y 0.822        z 0.5087       occ H    1 beq =beq3*1.5;
                    site H9a    x 0.448        y 0.75         z 0.5481       occ H    1 beq =beq3*1.5;
                    site H10a   x 0.486        y 0.75         z 0.6277       occ H    1 beq =beq3*1.5;
                    macro Stephens_monoclinic(s400, s040, s004, s220, s202, s022, s301, s121, s103, eta)
{
prm mhkl = H^4 s400 + K^4 s040 + L^4 s004 +
H^2 K^2 s220 + H^2 L^2 s202 + K^2 L^2 s022 +
H K^2 L s121 +
```

```
H L^3 s103 + H^3 L s301;

prm pp = D_spacing^2 * Sqrt(Max(mhkl,0)) / 1000;

gauss_fwhm = 1.8/3.1415927 pp (1-eta) Tan(Th) + 0.0001;
lor_fwhm = 1.8/3.1415927 pp eta Tan(Th) + 0.0001;
}
                    Out_CIF_STR("m-anis2cucl4_stephens.cif")
                    CS_G(@, 241.76058`_1.51137)
                    Strain_L(@, 0.05087`_0.00279)
                    Strain_G(@, 0.18646`_0.00319)
                    Preferred_Orientation(@, 0.55695`_0.00113,, 0 0 2)
                    Preferred_Orientation(@, 0.65230`_0.00214,, 2 0 0)
                    prm s400 -1339.62031`_97.90519
                    prm s040 -544.80559`_32.38638
                    prm s004 -11.32564`_0.60929
                    prm s220  1585.08574`_100.86239
                    prm s202  201.24689`_14.41267
                    prm s022  294.44379`_13.88905
                    prm s301  491.62432`_33.11644
                    prm s121  97.61912`_27.00700
                    prm s103 -43.17625`_2.02332
                    prm eta  0.00000`_0.03558_LIMIT_MIN_0 min 0 max 1
                    Stephens_monoclinic(s400, s040, s004, s220, s202, s022, s301, s121, s103, eta)
```

Final refinement stage (Stacking faults approach)

```
iters 20000
xdd maniscucl4.xye
   macro refine_layer_a      { @ }
   macro refine_layer_b      { @ }
   macro refine_layer_c      {  }

temperature 0.7
temperature 1.2
continue_after_convergence


          r_wp  13.7289401 r_exp  2.4014722 r_p  9.12886407 r_wp_dash  23.404778 r_p_dash  19.7478365 r_exp_dash  4.09397398
weighted_Durbin_Watson  0.0642909628 gof  5.71688489
   lam
          ymin_on_ymax 0.0001
      la 1 lo 1.540596 lh 0.5

          bkg  @  977.567406` -657.82872`  114.465665` -33.6831665`  127.209969` -153.079847`  39.7727203`  97.6117976` -
99.7988559`  58.603463`

          Radius(240)

          Full_Axial_Model(12, 15, 12, 2.3, @  2.95207`)

          Zero_Error(@, 0.00354`)
          LP_Factor(26.4)
          str
                    CS_G(@, 212.22563`)

                    CS_L(@, 9333.71855`)
                    Strain_L(@, 0.00015`)
                    Strain_G(@, 0.18754`)
                    scale @  6.6148515e-008`
                    prm c_sf  31.42763` min 31.0 max 32
                    space_group "P1"
                    a @  8.104102` min 8 max 8.2
                    b @  7.335897` min 7.2 max 7.4
                    c =c_sf*Nc;
                    al 90
                    be @  96.71076` min 96.6 max 96.8
                    ga 90
                    volume  92779.944`
                    macro XX( x) { x }
      macro YY( y) { y }
      macro ZZ(& z) { = z / Nc; }
      prm beq1  1.36063` min 0.2
                    prm beq2  0.63479` min 0.2
                    prm beq3  0.20000` min 0.2
                    Preferred_Orientation(@, 0.58203`,, 0 0 2)
                    Preferred_Orientation(@, 0.71224`,, 2 0 0)
      load site x y z occ beq layer {
                     Cu1_1  XX( 0.7520   )     YY( 0.8482  )      ZZ( 0.74972   )  Cu   1 =beq1;   A
                     Cu1_2  XX( -0.7520  )     YY( 1.3482  )      ZZ( -0.24972  )  Cu   1 =beq1;   A
                     Cu1_3  XX( -0.7520  )     YY( -0.8482 )      ZZ( -0.74972  )  Cu   1 =beq1;   A
                     Cu1_4  XX( 0.7520   )     YY( -0.3482 )      ZZ( 1.24972   )  Cu   1 =beq1;   A
                     Cl1_1  XX( 0.9390   )     YY( 1.0900  )      ZZ( 0.76261   )  Cl   1 =beq2;   A
                     Cl1_2  XX( -0.9390  )     YY( 1.5900  )      ZZ( -0.26261  )  Cl   1 =beq2;   A
                     Cl1_3  XX( -0.9390  )     YY( -1.0900 )      ZZ( -0.76261  )  Cl   1 =beq2;   A
                     Cl1_4  XX( 0.9390   )     YY( -0.5900 )      ZZ( 1.26261   )  Cl   1 =beq2;   A
                     Cl2_1  XX( 0.7254   )     YY( 0.9111  )      ZZ( 0.67932   )  Cl   1 =beq2;   A
                     Cl2_2  XX( -0.7254  )     YY( 1.4111  )      ZZ( -0.17932  )  Cl   1 =beq2;   A
                     Cl2_3  XX( -0.7254  )     YY( -0.9111 )      ZZ( -0.67932  )  Cl   1 =beq2;   A
                     Cl2_4  XX( 0.7254   )     YY( -0.4111 )      ZZ( 1.17932   )  Cl   1 =beq2;   A
                     Cl3_1  XX( 0.5649   )     YY( 0.6063  )      ZZ( 0.73684   )  Cl   1 =beq2;   A
                     Cl3_2  XX( -0.5649  )     YY( 1.1063  )      ZZ( -0.23684  )  Cl   1 =beq2;   A
                     Cl3_3  XX( -0.5649  )     YY( -0.6063 )      ZZ( -0.73684  )  Cl   1 =beq2;   A
                     Cl3_4  XX( 0.5649   )     YY( -0.1063 )      ZZ( 1.23684   )  Cl   1 =beq2;   A
                     Cl4_1  XX( 0.7785   )     YY( 0.7852  )      ZZ( 0.82013   )  Cl   1 =beq2;   A
                     Cl4_2  XX( -0.7785  )     YY( 1.2852  )      ZZ( -0.32013  )  Cl   1 =beq2;   A
                     Cl4_3  XX( -0.7785  )     YY( -0.7852 )      ZZ( -0.82013  )  Cl   1 =beq2;   A
                     Cl4_4  XX( 0.7785   )     YY( -0.2852 )      ZZ( 1.32013   )  Cl   1 =beq2;   A
                     O1_1   XX( 0.391    )     YY( 0.21    )      ZZ( 0.0478    )  O    1 =beq3;   A
                     O1_2   XX( -0.391   )     YY( 0.71    )      ZZ( 0.4522    )  O    1 =beq3;   A
```

```
O1_3   XX( -0.391   )     YY( -0.21    )      ZZ( -0.0478   )  O     1 =beq3;   A
O1_4   XX( 0.391    )     YY( 0.29     )      ZZ( 0.5478    )  O     1 =beq3;   A
N1_1   XX( 0.765    )     YY( 0.120    )      ZZ( 0.1748    )  N     1 =beq3;   A
N1_2   XX( -0.765   )     YY( 0.620    )      ZZ( 0.3252    )  N     1 =beq3;   A
N1_3   XX( -0.765   )     YY( -0.120   )      ZZ( -0.1748   )  N     1 =beq3;   A
N1_4   XX( 0.765    )     YY( 0.380    )      ZZ( 0.6748    )  N     1 =beq3;   A
C1_1   XX( 0.7355   )     YY( 0.120    )      ZZ( 0.1323    )  C     1 =beq3;   A
C1_2   XX( -0.7355  )     YY( 0.620    )      ZZ( 0.3677    )  C     1 =beq3;   A
C1_3   XX( -0.7355  )     YY( -0.120   )      ZZ( -0.1323   )  C     1 =beq3;   A
C1_4   XX( 0.7355   )     YY( 0.380    )      ZZ( 0.6323    )  C     1 =beq3;   A
C2_1   XX( 0.578    )     YY( 0.164    )      ZZ( 0.1122    )  C     1 =beq3;   A
C2_2   XX( -0.578   )     YY( 0.664    )      ZZ( 0.3878    )  C     1 =beq3;   A
C2_3   XX( -0.578   )     YY( -0.164   )      ZZ( -0.1122   )  C     1 =beq3;   A
C2_4   XX( 0.578    )     YY( 0.336    )      ZZ( 0.6122    )  C     1 =beq3;   A
C3_1   XX( 0.547    )     YY( 0.164    )      ZZ( 0.0676    )  C     1 =beq3;   A
C3_2   XX( -0.547   )     YY( 0.664    )      ZZ( 0.4324    )  C     1 =beq3;   A
C3_3   XX( -0.547   )     YY( -0.164   )      ZZ( -0.0676   )  C     1 =beq3;   A
C3_4   XX( 0.547    )     YY( 0.336    )      ZZ( 0.5676    )  C     1 =beq3;   A
C4_1   XX( 0.6742   )     YY( 0.120    )      ZZ( 0.0430    )  C     1 =beq3;   A
C4_2   XX( -0.6742  )     YY( 0.620    )      ZZ( 0.4570    )  C     1 =beq3;   A
C4_3   XX( -0.6742  )     YY( -0.120   )      ZZ( -0.0430   )  C     1 =beq3;   A
C4_4   XX( 0.6742   )     YY( 0.380    )      ZZ( 0.5430    )  C     1 =beq3;   A
C5_1   XX( 0.832    )     YY( 0.076    )      ZZ( 0.0631    )  C     1 =beq3;   A
C5_2   XX( -0.832   )     YY( 0.576    )      ZZ( 0.4369    )  C     1 =beq3;   A
C5_3   XX( -0.832   )     YY( -0.076   )      ZZ( -0.0631   )  C     1 =beq3;   A
C5_4   XX( 0.832    )     YY( 0.424    )      ZZ( 0.5631    )  C     1 =beq3;   A
C6_1   XX( 0.863    )     YY( 0.076    )      ZZ( 0.1077    )  C     1 =beq3;   A
C6_2   XX( -0.863   )     YY( 0.576    )      ZZ( 0.3923    )  C     1 =beq3;   A
C6_3   XX( -0.863   )     YY( -0.076   )      ZZ( -0.1077   )  C     1 =beq3;   A
C6_4   XX( 0.863    )     YY( 0.424    )      ZZ( 0.6077    )  C     1 =beq3;   A
C7_1   XX( 0.272    )     YY( 0.25     )      ZZ( 0.0698    )  C     1 =beq3;   A
C7_2   XX( -0.272   )     YY( 0.75     )      ZZ( 0.4302    )  C     1 =beq3;   A
C7_3   XX( -0.272   )     YY( -0.25    )      ZZ( -0.0698   )  C     1 =beq3;   A
C7_4   XX( 0.272    )     YY( 0.25     )      ZZ( 0.5698    )  C     1 =beq3;   A
H1_1   XX( 0.83     )     YY( 0.036    )      ZZ( 0.1877    )  H     1 =beq3*1.5;   A
H1_2   XX( -0.83    )     YY( 0.536    )      ZZ( 0.3123    )  H     1 =beq3*1.5;   A
H1_3   XX( -0.83    )     YY( -0.036   )      ZZ( -0.1877   )  H     1 =beq3*1.5;   A
H1_4   XX( 0.83     )     YY( 0.464    )      ZZ( 0.6877    )  H     1 =beq3*1.5;   A
H2_1   XX( 0.81     )     YY( 0.212    )      ZZ( 0.1879    )  H     1 =beq3*1.5;   A
H2_2   XX( -0.81    )     YY( 0.712    )      ZZ( 0.3121    )  H     1 =beq3*1.5;   A
H2_3   XX( -0.81    )     YY( -0.212   )      ZZ( -0.1879   )  H     1 =beq3*1.5;   A
H2_4   XX( 0.81     )     YY( 0.288    )      ZZ( 0.6879    )  H     1 =beq3*1.5;   A
H3_1   XX( 0.683    )     YY( 0.11     )      ZZ( 0.1900    )  H     1 =beq3*1.5;   A
H3_2   XX( -0.683   )     YY( 0.61     )      ZZ( 0.3100    )  H     1 =beq3*1.5;   A
H3_3   XX( -0.683   )     YY( -0.11    )      ZZ( -0.1900   )  H     1 =beq3*1.5;   A
H3_4   XX( 0.683    )     YY( 0.39     )      ZZ( 0.6900    )  H     1 =beq3*1.5;   A
H4_1   XX( 0.24     )     YY( 0.39     )      ZZ( 0.075     )  H     1 =beq3*1.5;   A
H4_2   XX( -0.24    )     YY( 0.89     )      ZZ( 0.425     )  H     1 =beq3*1.5;   A
H4_3   XX( -0.24    )     YY( -0.39    )      ZZ( -0.075    )  H     1 =beq3*1.5;   A
H4_4   XX( 0.24     )     YY( 0.11     )      ZZ( 0.575     )  H     1 =beq3*1.5;   A
H5_1   XX( 0.144    )     YY( 0.20     )      ZZ( 0.059     )  H     1 =beq3*1.5;   A
H5_2   XX( -0.144   )     YY( 0.70     )      ZZ( 0.441     )  H     1 =beq3*1.5;   A
H5_3   XX( -0.144   )     YY( -0.20    )      ZZ( -0.059    )  H     1 =beq3*1.5;   A
H5_4   XX( 0.144    )     YY( 0.30     )      ZZ( 0.559     )  H     1 =beq3*1.5;   A
H6_1   XX( 0.277    )     YY( 0.20     )      ZZ( 0.1037    )  H     1 =beq3*1.5;   A
H6_2   XX( -0.277   )     YY( 0.70     )      ZZ( 0.3963    )  H     1 =beq3*1.5;   A
H6_3   XX( -0.277   )     YY( -0.20    )      ZZ( -0.1037   )  H     1 =beq3*1.5;   A
H6_4   XX( 0.277    )     YY( 0.30     )      ZZ( 0.6037    )  H     1 =beq3*1.5;   A
H7_1   XX( 0.478    )     YY( 0.198    )      ZZ( 0.1314    )  H     1 =beq3*1.5;   A
H7_2   XX( -0.478   )     YY( 0.698    )      ZZ( 0.3686    )  H     1 =beq3*1.5;   A
H7_3   XX( -0.478   )     YY( -0.198   )      ZZ( -0.1314   )  H     1 =beq3*1.5;   A
H7_4   XX( 0.478    )     YY( 0.302    )      ZZ( 0.6314    )  H     1 =beq3*1.5;   A
H8_1   XX( 0.6502   )     YY( 0.120    )      ZZ( 0.0081    )  H     1 =beq3*1.5;   A
H8_2   XX( -0.6502  )     YY( 0.620    )      ZZ( 0.4919    )  H     1 =beq3*1.5;   A
H8_3   XX( -0.6502  )     YY( -0.120   )      ZZ( -0.0081   )  H     1 =beq3*1.5;   A
H8_4   XX( 0.6502   )     YY( 0.380    )      ZZ( 0.5081    )  H     1 =beq3*1.5;   A
H9_1   XX( 0.931    )     YY( 0.042    )      ZZ( 0.0439    )  H     1 =beq3*1.5;   A
H9_2   XX( -0.931   )     YY( 0.542    )      ZZ( 0.4561    )  H     1 =beq3*1.5;   A
H9_3   XX( -0.931   )     YY( -0.042   )      ZZ( -0.0439   )  H     1 =beq3*1.5;   A
H9_4   XX( 0.931    )     YY( 0.458    )      ZZ( 0.5439    )  H     1 =beq3*1.5;   A
H10_1  XX( 0.986    )     YY( 0.042    )      ZZ( 0.1234    )  H     1 =beq3*1.5;   A
H10_2  XX( -0.986   )     YY( 0.542    )      ZZ( 0.3766    )  H     1 =beq3*1.5;   A
H10_3  XX( -0.986   )     YY( -0.042   )      ZZ( -0.1234   )  H     1 =beq3*1.5;   A
H10_4  XX( 0.986    )     YY( 0.458    )      ZZ( 0.6234    )  H     1 =beq3*1.5;   A
O1a_1  XX( -0.101   )     YY( 0.90     )      ZZ( 0.5443    )  O     1 =beq3;   A
O1a_2  XX( 0.101    )     YY( 1.40     )      ZZ( -0.0443   )  O     1 =beq3;   A
O1a_3  XX( 0.101    )     YY( -0.90    )      ZZ( -0.5443   )  O     1 =beq3;   A
O1a_4  XX( -0.101   )     YY( -0.40    )      ZZ( 1.0443    )  O     1 =beq3;   A
N1a_1  XX( 0.254    )     YY( 0.822    )      ZZ( 0.6821    )  N     1 =beq3;   A
N1a_2  XX( -0.254   )     YY( 1.322    )      ZZ( -0.1821   )  N     1 =beq3;   A
N1a_3  XX( -0.254   )     YY( -0.822   )      ZZ( -0.6821   )  N     1 =beq3;   A
N1a_4  XX( 0.254    )     YY( -0.322   )      ZZ( 1.1821    )  N     1 =beq3;   A
C1a_1  XX( 0.2306   )     YY( 0.822    )      ZZ( 0.6331    )  C     1 =beq3;   A
C1a_2  XX( -0.2306  )     YY( 1.322    )      ZZ( -0.1331   )  C     1 =beq3;   A
C1a_3  XX( -0.2306  )     YY( -0.822   )      ZZ( -0.6331   )  C     1 =beq3;   A
C1a_4  XX( 0.2306   )     YY( -0.322   )      ZZ( 1.1331    )  C     1 =beq3;   A
C2a_1  XX( 0.075    )     YY( 0.860    )      ZZ( 0.6110    )  C     1 =beq3;   A
C2a_2  XX( -0.075   )     YY( 1.360    )      ZZ( -0.1110   )  C     1 =beq3;   A
C2a_3  XX( -0.075   )     YY( -0.860   )      ZZ( -0.6110   )  C     1 =beq3;   A
C2a_4  XX( 0.075    )     YY( -0.360   )      ZZ( 1.1110    )  C     1 =beq3;   A
C3a_1  XX( 0.053    )     YY( 0.860    )      ZZ( 0.5663    )  C     1 =beq3;   A
C3a_2  XX( -0.053   )     YY( 1.360    )      ZZ( -0.0663   )  C     1 =beq3;   A
C3a_3  XX( -0.053   )     YY( -0.860   )      ZZ( -0.5663   )  C     1 =beq3;   A
C3a_4  XX( 0.053    )     YY( -0.360   )      ZZ( 1.0663    )  C     1 =beq3;   A
C4a_1  XX( 0.1876   )     YY( 0.822    )      ZZ( 0.5436    )  C     1 =beq3;   A
C4a_2  XX( -0.1876  )     YY( 1.322    )      ZZ( -0.0436   )  C     1 =beq3;   A
C4a_3  XX( -0.1876  )     YY( -0.822   )      ZZ( -0.5436   )  C     1 =beq3;   A
C4a_4  XX( 0.1876   )     YY( -0.322   )      ZZ( 1.0436    )  C     1 =beq3;   A
C5a_1  XX( 0.343    )     YY( 0.783    )      ZZ( 0.5657    )  C     1 =beq3;   A
C5a_2  XX( -0.343   )     YY( 1.283    )      ZZ( -0.0657   )  C     1 =beq3;   A
C5a_3  XX( -0.343   )     YY( -0.783   )      ZZ( -0.5657   )  C     1 =beq3;   A
C5a_4  XX( 0.343    )     YY( -0.283   )      ZZ( 1.0657    )  C     1 =beq3;   A
```

```
C6a_1  XX( 0.365    )      YY( 0.783   )       ZZ( 0.6105    )  C    1 =beq3;   A
C6a_2  XX( -0.365   )      YY( 1.283   )       ZZ( -0.1105   )  C    1 =beq3;   A
C6a_3  XX( -0.365   )      YY( -0.783  )       ZZ( -0.6105   )  C    1 =beq3;   A
C6a_4  XX( 0.365    )      YY( -0.283  )       ZZ( 1.1105    )  C    1 =beq3;   A
C7a_1  XX( -0.238   )      YY( 0.93    )       ZZ( 0.5660    )  C    1 =beq3;   A
C7a_2  XX( 0.238    )      YY( 1.43    )       ZZ( -0.0660   )  C    1 =beq3;   A
C7a_3  XX( 0.238    )      YY( -0.93   )       ZZ( -0.5660   )  C    1 =beq3;   A
C7a_4  XX( -0.238   )      YY( -0.43   )       ZZ( 1.0660    )  C    1 =beq3;   A
H1a_1  XX( 0.191    )      YY( 0.888   )       ZZ( 0.6960    )  H    1 =beq3*1.5;   A
H1a_2  XX( -0.191   )      YY( 1.388   )       ZZ( -0.1960   )  H    1 =beq3*1.5;   A
H1a_3  XX( -0.191   )      YY( -0.888  )       ZZ( -0.6960   )  H    1 =beq3*1.5;   A
H1a_4  XX( 0.191    )      YY( -0.388  )       ZZ( 1.1960    )  H    1 =beq3*1.5;   A
H2a_1  XX( 0.24     )      YY( 0.722   )       ZZ( 0.6959    )  H    1 =beq3*1.5;   A
H2a_2  XX( -0.24    )      YY( 1.222   )       ZZ( -0.1959   )  H    1 =beq3*1.5;   A
H2a_3  XX( -0.24    )      YY( -0.722  )       ZZ( -0.6959   )  H    1 =beq3*1.5;   A
H2a_4  XX( 0.24     )      YY( -0.222  )       ZZ( 1.1959    )  H    1 =beq3*1.5;   A
H3a_1  XX( 0.347    )      YY( 0.86    )       ZZ( 0.6957    )  H    1 =beq3*1.5;   A
H3a_2  XX( -0.347   )      YY( 1.36    )       ZZ( -0.1957   )  H    1 =beq3*1.5;   A
H3a_3  XX( -0.347   )      YY( -0.86   )       ZZ( -0.6957   )  H    1 =beq3*1.5;   A
H3a_4  XX( 0.347    )      YY( -0.36   )       ZZ( 1.1957    )  H    1 =beq3*1.5;   A
H4a_1  XX( -0.343   )      YY( 0.84    )       ZZ( 0.564     )  H    1 =beq3*1.5;   A
H4a_2  XX( 0.343    )      YY( 1.34    )       ZZ( -0.064    )  H    1 =beq3*1.5;   A
H4a_3  XX( 0.343    )      YY( -0.84   )       ZZ( -0.564    )  H    1 =beq3*1.5;   A
H4a_4  XX( -0.343   )      YY( -0.34   )       ZZ( 1.064     )  H    1 =beq3*1.5;   A
H5a_1  XX( -0.221   )      YY( 0.95    )       ZZ( 0.6014    )  H    1 =beq3*1.5;   A
H5a_2  XX( 0.221    )      YY( 1.45    )       ZZ( -0.1014   )  H    1 =beq3*1.5;   A
H5a_3  XX( 0.221    )      YY( -0.95   )       ZZ( -0.6014   )  H    1 =beq3*1.5;   A
H5a_4  XX( -0.221   )      YY( -0.45   )       ZZ( 1.1014    )  H    1 =beq3*1.5;   A
H6a_1  XX( -0.31    )      YY( 1.06    )       ZZ( 0.559     )  H    1 =beq3*1.5;   A
H6a_2  XX( 0.31     )      YY( 1.56    )       ZZ( -0.059    )  H    1 =beq3*1.5;   A
H6a_3  XX( 0.31     )      YY( -1.06   )       ZZ( -0.559    )  H    1 =beq3*1.5;   A
H6a_4  XX( -0.31    )      YY( -0.56   )       ZZ( 1.059     )  H    1 =beq3*1.5;   A
H7a_1  XX( -0.030   )      YY( 0.89    )       ZZ( 0.6286    )  H    1 =beq3*1.5;   A
H7a_2  XX( 0.030    )      YY( 1.39    )       ZZ( -0.1286   )  H    1 =beq3*1.5;   A
H7a_3  XX( 0.030    )      YY( -0.89   )       ZZ( -0.6286   )  H    1 =beq3*1.5;   A
H7a_4  XX( -0.030   )      YY( -0.39   )       ZZ( 1.1286    )  H    1 =beq3*1.5;   A
H8a_1  XX( 0.171    )      YY( 0.822   )       ZZ( 0.5087    )  H    1 =beq3*1.5;   A
H8a_2  XX( -0.171   )      YY( 1.322   )       ZZ( -0.0087   )  H    1 =beq3*1.5;   A
H8a_3  XX( -0.171   )      YY( -0.822  )       ZZ( -0.5087   )  H    1 =beq3*1.5;   A
H8a_4  XX( 0.171    )      YY( -0.322  )       ZZ( 1.0087    )  H    1 =beq3*1.5;   A
H9a_1  XX( 0.448    )      YY( 0.75    )       ZZ( 0.5481    )  H    1 =beq3*1.5;   A
H9a_2  XX( -0.448   )      YY( 1.25    )       ZZ( -0.0481   )  H    1 =beq3*1.5;   A
H9a_3  XX( -0.448   )      YY( -0.75   )       ZZ( -0.5481   )  H    1 =beq3*1.5;   A
H9a_4  XX( 0.448    )      YY( -0.25   )       ZZ( 1.0481    )  H    1 =beq3*1.5;   A
H10a_1 XX( 0.486    )      YY( 0.75    )       ZZ( 0.6277    )  H    1 =beq3*1.5;   A
H10a_2 XX( -0.486   )      YY( 1.25    )       ZZ( -0.1277   )  H    1 =beq3*1.5;   A
H10a_3 XX( -0.486   )      YY( -0.75   )       ZZ( -0.6277   )  H    1 =beq3*1.5;   A
H10a_4 XX( 0.486    )      YY( -0.25   )       ZZ( 1.1277    )  H    1 =beq3*1.5;   A
}

            macro slojA(ze, sxc, sxv, syc, syv, szc, szv)
   {
      #m_argu sxc
      #m_argu syc
      #m_argu szc
      If_Prm_Eqn_Rpt(szc, szv, min = -0.25; max = 0.25; val_on_continue = Rand(-0.25,0.25);)

      stack A
         sx sxc sxv randomize(a)
         sy syc syv randomize(b)
         sz = (ze + CeV(szc, szv)) / Nc;

         #ifdef generiraj_placeholdere
            #m_ifarg ze "0" #m_else
               generate_these "*"
                  generate_name_append _##ze
            #m_endif
         #endif

   }
         macro slojB(ze, sxc, sxv, syc, syv, szc, szv)
   {
      #m_argu sxc
      #m_argu syc
      #m_argu szc
      If_Prm_Eqn_Rpt(szc, szv, min = -0.25; max = 0.25; val_on_continue = Rand(-0.25,0.25);)

      stack B
         sx sxc sxv randomize(a)
         sy syc syv randomize(b)
         sz = (ze + CeV(szc, szv)) / Nc;

         #ifdef generiraj_placeholdere
            #m_ifarg ze "0" #m_else
               generate_these "*"
                  generate_name_append _##ze
            #m_endif
         #endif

   }

prm !Nc 50

slojA(0,   ,0, ,0, ,0)
slojA(1,  refine_layer_a, 0.00000`, refine_layer_b, 0.00000`, refine_layer_c,0)
slojA(2,  refine_layer_a, 0.00073`, refine_layer_b, 0.00000`, refine_layer_c,0)
slojA(3,  refine_layer_a, 0.99984`, refine_layer_b, 0.99081`, refine_layer_c,0)
slojA(4,  refine_layer_a, 0.99985`, refine_layer_b, 0.98946`, refine_layer_c,0)
slojA(5,  refine_layer_a, 0.99945`, refine_layer_b, 0.00000`, refine_layer_c,0)
slojA(6,  refine_layer_a, 0.00000`, refine_layer_b, 0.00000`, refine_layer_c,0)
slojA(7,  refine_layer_a, 0.99183`, refine_layer_b, 0.98592`, refine_layer_c,0)
slojA(8,  refine_layer_a, 0.98552`, refine_layer_b, 0.98644`, refine_layer_c,0)
```

```
slojA(9,  refine_layer_a, 0.97805`, refine_layer_b, 0.00000`, refine_layer_c,0)
slojA(10, refine_layer_a, 0.97197`, refine_layer_b, 0.00000`, refine_layer_c,0)
slojA(11, refine_layer_a, 0.96716`, refine_layer_b, 0.00000`, refine_layer_c,0)
slojA(12, refine_layer_a, 0.96588`, refine_layer_b, 0.00000`, refine_layer_c,0)
slojA(13, refine_layer_a, 0.96684`, refine_layer_b, 0.99990`, refine_layer_c,0)
slojA(14, refine_layer_a, 0.97229`, refine_layer_b, 0.99990`, refine_layer_c,0)
slojA(15, refine_layer_a, 0.97584`, refine_layer_b, 0.99990`, refine_layer_c,0)
slojA(16, refine_layer_a, 0.97281`, refine_layer_b, 0.99990`, refine_layer_c,0)
slojA(17, refine_layer_a, 0.97335`, refine_layer_b, 0.41028`, refine_layer_c,0)
slojA(18, refine_layer_a, 0.97390`, refine_layer_b, 0.39117`, refine_layer_c,0)
slojA(19, refine_layer_a, 0.97046`, refine_layer_b, 0.41365`, refine_layer_c,0)
slojA(20, refine_layer_a, 0.98479`, refine_layer_b, 0.46551`, refine_layer_c,0)
slojA(20, refine_layer_a, 0.51697`, refine_layer_b, 0.00000`, refine_layer_c,0)
slojA(21, refine_layer_a, 0.50418`, refine_layer_b, 0.01731`, refine_layer_c,0)
slojA(22, refine_layer_a, 0.99054`, refine_layer_b, 0.99990`, refine_layer_c,0)
slojA(23, refine_layer_a, 0.95552`, refine_layer_b, 0.99990`, refine_layer_c,0)
slojA(24, refine_layer_a, 0.97802`, refine_layer_b, 0.02260`, refine_layer_c,0)
slojA(25, refine_layer_a, 0.97915`, refine_layer_b, 0.01966`, refine_layer_c,0)
slojA(26, refine_layer_a, 0.98585`, refine_layer_b, 0.99990`, refine_layer_c,0)
slojA(27, refine_layer_a, 0.98435`, refine_layer_b, 0.99990`, refine_layer_c,0)
slojA(28, refine_layer_a, 0.98821`, refine_layer_b, 0.00000`, refine_layer_c,0)
slojA(29, refine_layer_a, 0.99010`, refine_layer_b, 0.00000`, refine_layer_c,0)
slojA(30, refine_layer_a, 0.99171`, refine_layer_b, 0.00000`, refine_layer_c,0)
slojA(31, refine_layer_a, 0.00000`, refine_layer_b, 0.00000`, refine_layer_c,0)
slojA(32, refine_layer_a, 0.98633`, refine_layer_b, 0.82676`, refine_layer_c,0)
slojA(33, refine_layer_a, 0.98273`, refine_layer_b, 0.77586`, refine_layer_c,0)
slojA(34, refine_layer_a, 0.98120`, refine_layer_b, 0.74314`, refine_layer_c,0)
slojA(35, refine_layer_a, 0.98128`, refine_layer_b, 0.75978`, refine_layer_c,0)
slojA(36, refine_layer_a, 0.97951`, refine_layer_b, 0.79191`, refine_layer_c,0)
slojA(37, refine_layer_a, 0.98379`, refine_layer_b, 0.00000`, refine_layer_c,0)
slojA(38, refine_layer_a, 0.98603`, refine_layer_b, 0.00000`, refine_layer_c,0)
slojA(39, refine_layer_a, 0.99106`, refine_layer_b, 0.99990`, refine_layer_c,0)
slojA(40, refine_layer_a, 0.99412`, refine_layer_b, 0.03186`, refine_layer_c,0)
slojA(41, refine_layer_a, 0.00000`, refine_layer_b, 0.03430`, refine_layer_c,0)
slojA(42, refine_layer_a, 0.99126`, refine_layer_b, 0.04208`, refine_layer_c,0)
slojA(43, refine_layer_a, 0.98990`, refine_layer_b, 0.99990`, refine_layer_c,0)
slojA(44, refine_layer_a, 0.98609`, refine_layer_b, 0.99990`, refine_layer_c,0)
slojA(45, refine_layer_a, 0.98190`, refine_layer_b, 0.99990`, refine_layer_c,0)
slojA(46, refine_layer_a, 0.98016`, refine_layer_b, 0.05703`, refine_layer_c,0)
slojA(47, refine_layer_a, 0.98492`, refine_layer_b, 0.04939`, refine_layer_c,0)
slojA(48, refine_layer_a, 0.99115`, refine_layer_b, 0.05373`, refine_layer_c,0)
slojA(49, refine_layer_a, 0.98608`, refine_layer_b, 0.99990`, refine_layer_c,0)
macro LP(& l0, l)
   {
      AA l
      min = l0 - .1;
      max = l0 + .1;
   }
macro LPA(& l0, l)
   {
      AA l
      min = l0 - 2;
      max = l0 + 2;
   }
macro randomize(a)
   {
      min = 0;
      max = 0.9999;
      val_on_continue = Val + Rand(0, 1) T / Constant(Get(a));
   }
```

Results and discussion

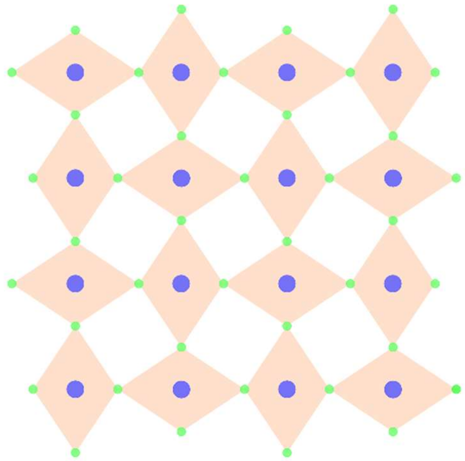

Figure S10. Antiferrodistortive arrangement of $CuCl_4$ units (distortions are exaggerated).

Table S4. Hydrogen bond geometry in the crystal structures of **(*o*-A)$_2$CuCl$_4$**, **(*m*-A)$_2$CuCl$_4$**, **(*p*-A)$_2$CuCl$_4$** and **(*m*-A*)$_2$CuCl$_4$**.

**(*o*-A)$_2$CuCl$_4$**					
D–H···A	ARU	*d*(D–H)/Å	*d*(H···A)/Å	*d*(D···A)/Å	∠(D–H···A)/°
N1–H1A···Cl2	1-x,1-y,1-z	0.87(2)	2.40(2)	3.2591(17)	169(3)
N1–H1B···Cl1	x,y,z	0.85(2)	2.43(2)	3.2341(16)	158(2)
N1–H1C···Cl1	3/2-x,1/2+y,3/2-z	0.84(2)	2.75(2)	3.365(2)	131(2)
N1–H1C···Cl2	1/2+x,1/2-y,1/2+z	0.84(2)	2.71(3)	3.4668(18)	150(3)
**(*m*-A)$_2$CuCl$_4$**					
D–H···A	ARU	*d*(D–H)/Å	*d*(H···A)/Å	*d*(D···A)/Å	∠(D–H···A)/°
N1–H1···Cl1	2-x,1-y,1-z	0.88	2.47	3.3009(1)	157
N1A–H2A···Cl1	1-x,-1/2+y,3/2-z	0.87	2.28	3.0002(1)	141
N1–H3···Cl3	x,1/2-y,-1/2+z	0.87	2.44	3.1513(1)	140
N1A–H3A···Cl3	x,y,z	0.87	2.78	3.2864(1)	119
N1A–H3A···Cl3	1-x,1/2+y,3/2-z	0.87	2.81	3.4818(1)	135
**(*p*-A)$_2$CuCl$_4$**					
D–H···A	ARU	*d*(D–H)/Å	*d*(H···A)/Å	*d*(D···A)/Å	∠(D–H···A)/°
N1–H1A···Cl1	x,y,z	0.86(3)	2.38(3)	3.203(3)	161(5)
N1–H1B···Cl1	1/2-x,1/2+y,z	0.88(3)	2.43(2)	3.270(3)	162(5)
N1–H1C···Cl2	1/2-x,-1/2+y,z	0.88(5)	2.42(6)	3.287(3)	174(5)
**(*m*-A*)$_2$CuCl$_4$**					
D–H···A	ARU	*d*(D–H)/Å	*d*(H···A)/Å	*d*(D···A)/Å	∠(D–H···A)/°
N1–H1A···Cl1	x,1-y,-1/2+z	0.89	2.54	3.363(5)	155
N1–H1A···Cl2	x,1-y,-1/2+z	0.89	2.75	3.354(5)	126
N1–H1B···Cl2	3/2-x,-1/2+y,3/2-z	0.89	2.35	3.235(5)	176
N1–H1C···Cl1	x,y,z	0.89	2.41	3.233(5)	153

Dimensionality calculation

There are several published methods for the determination of the dimensionality of a crystal structure, the most prominent ones using the periodic net representation of the crystal structure,[1–3] determination of translational matrix rank (RDA)[4] and topology-scaling algorithm (TSA).[5] Even though the implementations of these algorithms give the correct dimensionality of the framework based on the selected bond length parameter, they do not discriminate between weakly and strongly connected *n*D systems. In order to distinguish between *n*D and almost-*n*D systems, we implemented a modification of the TSA algorithm as follows.

A supercell of $N_a \times N_b \times N_c$ cells is constructed. N is chosen to be at least 3 and such that six unit cell body diagonals can fit in the supercell. The reasoning behind this is to 1) have enough points to fit the dimensionality function, 2) limit the RAM requirements for calculation on highly elongated cells. A metal atom is chosen in a central cell, and a graph traversal algorithm is used to span a connected set of metal atoms with interatomic distances less than a selected threshold, t, which one can interpret as *interaction length*. Once the connected set is found, dimensionality, d_N, of the connected set is calculated by fitting the function:

$$N(atoms) \propto r^{d_N}$$

Furthermore, dimensionality d_N can be calculated as a function of t. From this, one can observe for which interaction length t the system becomes n-dimensional. This enables „quantification“ of dimensionality: if the dimensionality of the system is n at interaction length t, but becomes n' at a slightly higher interaction length, that system can be considered „almost-n'-dimensional“.

Finally, in order to compare structures with different inorganic constituents, we have normalized the parameter t according to sum of ionic radii[6] of metal and halogen ions representing the M-X-M bond ($2r_\mathrm{i}(\mathrm{M}) + 2r_\mathrm{i}(\mathrm{X})$), giving a normalized parameter t_n.

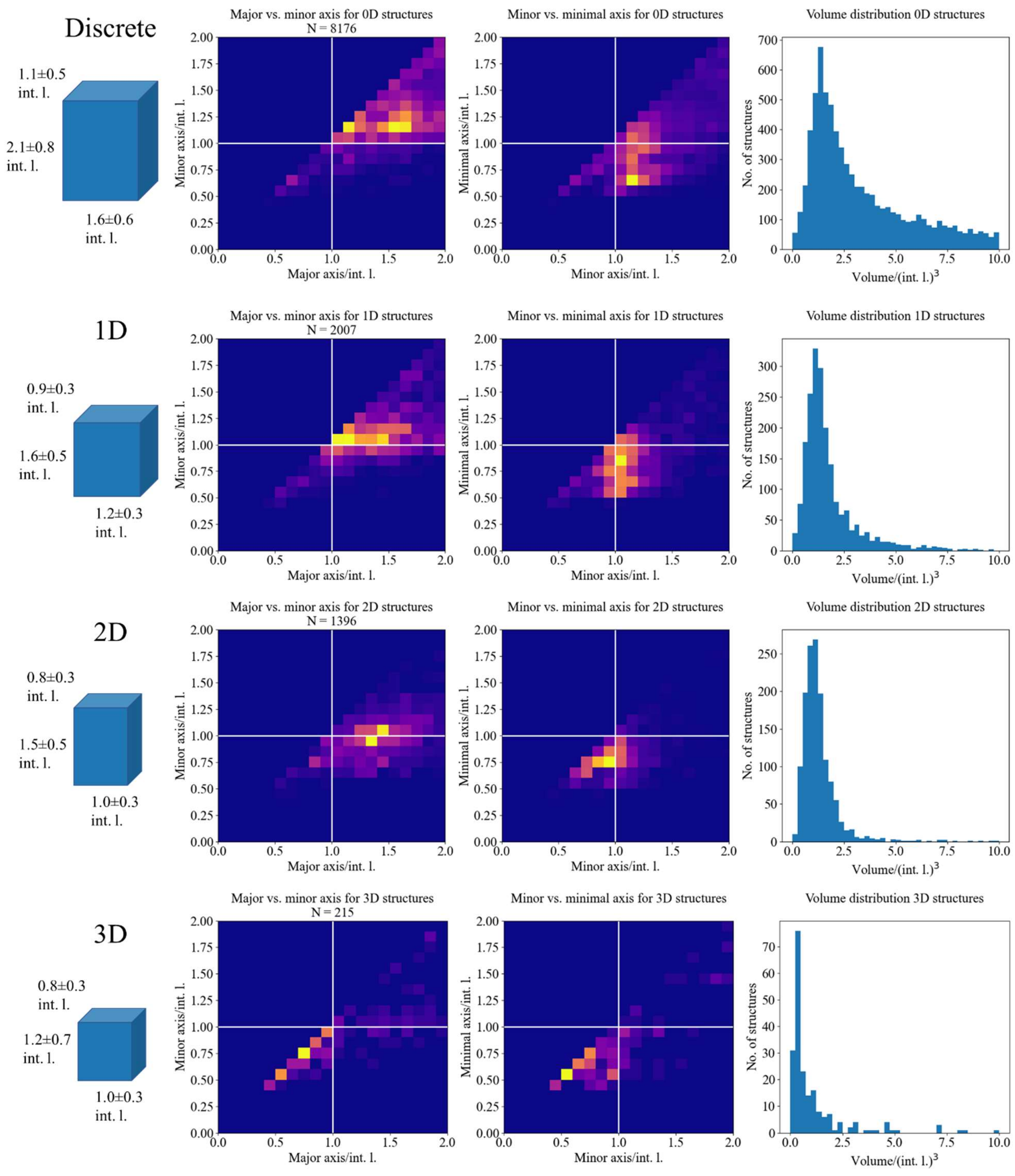

Figure S11. Organic cation bounding box dimension histograms for (top to bottom) discrete, 1D, 2D and 3D HOIHs. Figures represent (from left to right): the average bounding box, box major vs. minor axis distribution, box minor vs. minimal axis distribution, and box volume distribution. All dimensions are normalized to interaction length, i.e. the expected metal-metal distance $(2r_i(\mathrm{M}) + 2r_i(\mathrm{X}))$.

Python code for dimensionality calculation

```
#dependencies: cctbx, numpy, numba, scipy, tqdm,
import iotbx.cif
import glob
import os
from scitbx.array_family import flex
import numpy as np
from numba import njit
from scipy.spatial import distance
from matplotlib import pyplot as plt
from scipy.optimize import least_squares
from tqdm.contrib.concurrent import process_map
import pickle
from scipy.sparse import csr_matrix
from scipy.sparse.csgraph import breadth_first_order
from global_vars import datadir, datadir_acucl4, figdir

def generate_nodes(pts, a_range, b_range, c_range, mat):
#generate all metal nodes and find one that is in the central cell
    res = np.empty(shape=(len(pts) * (2 * a_range + 1) * (2 * b_range + 1) * (2 * c_range + 1), 3))
    cnt = 0
    center_node = 0
    for i in range(-a_range, a_range + 1):
        for j in range(-b_range, b_range + 1):
            for k in range(-c_range, c_range + 1):
                for p in pts:
                    res[cnt, 0] = p[0] + i
                    res[cnt, 1] = p[1] + j
                    res[cnt, 2] = p[2] + k
                    res[cnt] = np.matmul(mat, res[cnt])
                    cnt += 1
                    if i == 0 and j == 0 and k == 0 and center_node == 0:
                        center_node = cnt - 1
    return center_node, res


@njit
def calc_midpoint_distance(nodes, distances):
#calculate distances between nodes
    for i in range(nodes.shape[0]):
        for j in range(nodes.shape[0]):
            distances[i, j] = np.sqrt(
                (nodes[i][0] + nodes[j][0]) ** 2 + (nodes[i][1] + nodes[j][1]) ** 2 + (nodes[i][2] + nodes[j][2]) ** 2)
    return distances


def bfs_traversal(startnode, adj_matrix):
#return graph of connected set
    return breadth_first_order(csr_matrix(adj_matrix), startnode, return_predecessors=False)


@njit
def fit_func(x, t, y):
#helper function for extraction of dimensionality from N(r)
    return x[0] * np.power(t, x[1]) - y


def import_cif(filename_cif):
#import cif file and return metal elements, halogen element and fractional sites of metal atoms
    element_list = ['Li', 'Na', 'K', 'Rb', 'Cs', 'Be', 'Mg', 'Ca', 'Sr', 'Ba', 'Sc', 'Ti', 'V', 'Cr', 'Mn', 'Fe', 'Co',
                    'Ni', 'Cu', 'Zn', 'Al', 'Ga', 'In', 'Sn', 'Tl', 'Pb', 'Bi', 'Y', 'Zr', 'Nb', 'Mo', 'Tc', 'Ru', 'Rh',
                    'Pd', 'Ag', 'Cd', 'Hf', 'Ta', 'W', 'Re', 'Os', 'Ir', 'Pt', 'Au', 'Hg', 'La', 'Ce', 'Pr', 'Nd', 'Sm',
                    'Eu', 'Gd', 'Tb', 'Dy', 'Ho', 'Er', 'Tm', 'Yb', 'Lu', 'Ac', 'Th', 'Pa', 'U', 'Pu', 'Am', 'Ge', 'Sb']
    halogen_list = ['F', 'Cl', 'Br', 'I']
    struc = (
        iotbx.cif.reader(file_path=filename_cif)
        .build_crystal_structures()
        .popitem()[1]
    )
    struc.scattering_type_registry(table="xray")
    strucp1 = struc.expand_to_p1(sites_mod_positive=True)
    struct_sel = strucp1.select(flex.bool([sc.element_symbol() in element_list for sc in strucp1.scatterers()]))
    e_list = np.unique([sc.element_symbol() for sc in strucp1.scatterers()])
    return [x for x in e_list if (x in element_list)], [x for x in e_list if (x in halogen_list)], np.reshape(
        strucp1.unit_cell().orthogonalization_matrix(), (3, 3)), np.unique(
        struct_sel.sites_frac().as_numpy_array().round(8), axis=0)


def calculate_dimension(cif_file, min_threshold, max_threshold, threshold_step):
#calculate dimensionality as a function of threshold
    try:
        metals, halogens, mat, pts = import_cif(cif_file)
    except:
        return (['Error'], [0, 0])
    a_cell = float(mat[0, 0])
    b_cell = float(mat[1, 1])
    c_cell = float(mat[2, 2])
    vol = np.linalg.det(mat)
    available_ram_gb = 4
    corr_len = max(3 * np.power(vol, 1. / 3), max_threshold)
    expand = [max(int(np.ceil(corr_len / a_cell - 1)), 1), max(int(np.ceil(corr_len / b_cell - 1)), 1),
              max(int(np.ceil(corr_len / c_cell - 1)), 1)]
    #while (2 * expand[0] + 1) * (2 * expand[1] + 1) * (2 * expand[2] + 1) * len(pts) > np.sqrt(
    #        1024 * 1024 * 1024 * available_ram_gb / 64):
    #    expand[expand.index(max(expand))] -= 1
#find connected set and fit function N(r)
    center_node, nodes = generate_nodes(pts, expand[0], expand[1], expand[2], mat)
    dists = distance.cdist(nodes, nodes)
    np.fill_diagonal(dists, 10000.0)
```

```
    result = []
    for threshold in np.arange(min_threshold, max_threshold, threshold_step):
        framework = bfs_traversal(center_node, dists <= threshold)
        if len(framework) <= 1:
            result.append([np.round(threshold, 5), 0])
            continue
        center_of_nodes = np.average(nodes[framework], axis=0)
        local_dists = distance.cdist(nodes[framework], center_of_nodes[None, :])
        t = []
        y = []
        res_cntr = 0
        for i in np.arange(1, corr_len, 1, dtype=float):
            if res_cntr > 20:
                if y[res_cntr - 1] == y[res_cntr - int(threshold)]:
                    y = y[:-int(threshold)]
                    t = t[:-int(threshold)]
                    break
            tmp_cnt = np.count_nonzero(local_dists <= i)
            if tmp_cnt > 0:
                t.append(i)
                y.append(tmp_cnt)
                res_cntr += 1

        t = np.array(t, dtype=float)
        y = np.array(y, dtype=float)
        x0 = np.ones(2)
        res_robust = least_squares(fit_func, x0, args=(t, y))
        result.append([np.round(threshold, 5), np.round(res_robust.x[1], 5)])

    return ([metals, halogens], result)

def ionic_radius(str):
#helper function for ionic radii
    return {
        "F": 1.19,
        "Cl":1.81,
        "Br":1.82,
        "I":2.06,
        'Li':0.9,
        'Be':0.59,
        'Na':1.16,
        'Mg':0.86,
        'Al':0.675,
        'K':1.52,
        'Ca':1.14,
        'Sc':0.885,
        'Ti':1,
        'V':0.93,
        'Cr':0.94,
        'Mn':0.97,
        'Fe':0.92,
        'Co':0.885,
        'Ni':0.83,
        'Cu':0.91,
        'Zn':0.88,
        'Ga':0.76,
        'Ge':0.87,
        'Rb':1.66,
        'Sr':1.32,
        'Y':1.04,
        'Zr':0.86,
        'Nb':0.86,
        'Mo':0.83,
        'Tc':0.78,
        'Ru':0.82,
        'Rh':0.805,
        'Pd':1,
        'Ag':1.29,
        'Cd':1.09,
        'In':0.94,
        'Sn':1.18,
        'Sb':0.9,
        'Cs':1.67,
        'Ba':1.49,
        'La':1.172,
        'Ce':1.15,
        'Pr':1.13,
        'Nd':1.43,
        'Pm':1.11,
        'Sm':1.36,
        'Eu':1.31,
        'Gd':1.078,
        'Tb':1.063,
        'Dy':1.21,
        'Ho':1.041,
        'Er':1.03,
        'Tm':1.17,
        'Yb':1.16,
        'Lu':1.001,
        'Hf':0.71,
        'Ta':0.86,
        'W':0.66,
        'Re':0.63,
        'Os':0.63,
        'Ir':0.82,
        'Pt':0.94,
        'Au':1.51,
        'Hg':1.33,
        'Tl':1.64,
        'Pb':1.33,
        'Bi':1.17,
        'Fr':1.94,
```

```
        'Ra':1.62,
        'Ac':1.26,
        'Pa':1.16,
        'U':1.165,
        'Np':1.24,
        'Pu':1.14,
        'Am':1.4,
    }[str]


def try_calc_dim(file):
#helper function for parallelization
    return calculate_dimension(file, 3, 20, 0.05)

def calculate_from_cifs_in_folder(DIR=datadir):
#calculate all data and dump it in a pickled file, as well as text file
    os.chdir(DIR)
    files = [DIR + "/" + file for file in glob.glob("*.cif")]
    files = files[:100]
    outputs = process_map(try_calc_dim, files, chunksize=1, max_workers=8)

    with open(DIR + "/output.pickle", "wb") as kl:
        pickle.dump(outputs, kl)
    with open(DIR + "/results.txt", "w") as f:
        for file, output in zip(files, outputs):
            f.write(file + "#" + str(output) + "\n")

def plot_for_acucl4():
#plot dn(t) for anisidinium tetrachlorocuprates
    calculate_from_cifs_in_folder(datadir_acucl4)
    with open(datadir+"output.pickle", "rb") as f:
       outputs = pickle.load(f)
    plt.rcParams["font.family"] = "Times New Roman"
    plt.figure(1, figsize=[9.51, 5.91])
    plt.subplot(111, ylabel="Dimensionality", xlabel="Interaction length/(2$\it{r}$(Cu)+2$\it{r}$(Cl))")
    plt.gca().set_title("Dimensionality vs. interaction length for anisidinium tetrachlorocuprates")
    col = [(0, 0.5, 0), (0, 0, 0.5), (0.5, 0, 0)]
    for i, out in enumerate(outputs):
        pr = np.array(out[1])
        plt.plot(pr[:,0]/5.44, pr[:,1], color=col[i])
    plt.gca().xaxis.grid(True)
    plt.show()



def plot_all():
#plot bounding box parameters for each dimensionality
    with open(datadir+“output.pickle“, "rb") as f:
       outputs = pickle.load(f)
    bboxes = np.zeros(shape=(len(outputs),3))
    with open(datadir+“bb.vectors“, "r") as f:
        for i, line in enumerate(f.readlines()):
            b = line.split("#")[1].split(",")
            bboxes[i,0] = float(b[0])
            bboxes[i,1] = float(b[1])
            bboxes[i,2] = float(b[2])
    for j in [0, 1, 2, 3]:
        dims = []
        ars1 = []
        ars2 = []
        ars3 = []
        ars4 = []
        for i, out in enumerate(outputs):
            try:
                interaction_length = 2*ionic_radius(out[0][0][0])+2*ionic_radius(out[0][1][0])
            except:
                interaction_length = 5.0
            t = [x[1] for x in out[1] if x[0]==np.round(interaction_length,1)][0]
            if bboxes[i,0]>0 and t>=j-0.2 and t<j+0.2:
                dims.append(t)
                ars1.append(bboxes[i,0]/interaction_length)
                ars2.append(bboxes[i,1]/interaction_length)
                ars3.append(bboxes[i,2]/interaction_length)
                ars4.append(bboxes[i,0]*bboxes[i,1]*bboxes[i,2]/np.power(interaction_length,3))

        color_palette = plt.cm.plasma
        rng = [0, 2]
        rng2d = [rng, rng]
        plt.rcParams["font.family"] = "Times New Roman"
        plt.figure(1, figsize=[17.7,5.91])
        plt.subplot(131,ylabel="Minor axis/int. l.", xlabel="Major axis/int. l.")
        plt.gca().set_title("Major vs. minor axis for " + str(j) + "D structures, N = " + str(len(dims)))
        plt.hist2d(ars1,ars2,density=True, range=rng2d, bins=[20,20], cmap=color_palette)
        plt.plot([0, 2], [1, 1], 'k-', lw=2, color="white")
        plt.plot([1, 1], [0, 2], 'k-', lw=2, color="white")
        plt.subplot(132,ylabel="Minimal axis/int. l.", xlabel="Minor axis/int. l.")
        plt.gca().set_title("Minor vs. minimal axis for " + str(j) + "D structures")
        plt.hist2d(ars2,ars3,density=True, range=rng2d,bins=[20,20], cmap=color_palette)
        plt.plot([0, 2], [1, 1], 'k-', lw=2, color="white")
        plt.plot([1, 1], [0, 2], 'k-', lw=2, color="white")
        #plt.subplot(133,ylabel="Minimal axis/int. l.", xlabel="Major axis/int. l.")
        #plt.gca().set_title("Minor vs. minimal axis for " + str(j) + "D structures")
        #plt.hist2d(ars1,ars3,density=True, range=rng2d,bins=[20,20], cmap=color_palette)
        plt.subplot(133,ylabel="No. of structures", xlabel="Volume/(int. l.)$^3$")
        plt.gca().set_title("Volume distribution " + str(j) + "D structures")
        plt.hist(ars4,bins=40,range=[0,10])
        plt.savefig(figdir+"Figure_v_"+str(j)+".png")
        plt.clf()

def plot_all2():
#alternative plot
    with open(datadir+“output.pickle“, "rb") as f:
```

```
        outputs = pickle.load(f)
    bboxes = np.zeros(shape=(len(outputs),3))
    with open(datadir+"bb.vectors", "r") as f:
        for i, line in enumerate(f.readlines()):
            b = line.split("#")[1].split(",")
            bboxes[i,0] = float(b[0])
            bboxes[i,1] = float(b[1])
            bboxes[i,2] = float(b[2])
    for j in [1, 2, 3]:
        dims = []
        ars1 = []
        ars2 = []
        for i, out in enumerate(outputs):
            try:
                interaction_length = 2*ionic_radius(out[0][0][0])+2*ionic_radius(out[0][1][0])
            except:
                interaction_length = 5.0
            t = [x[0]/interaction_length for x in out[1] if np.isclose(np.round(x[1],0),j)]
            t.append(100)
            if bboxes[i,0]>0 and np.min(t)<100:
                dims.append(np.min(t))
                ars1.append(bboxes[i,0]*bboxes[i,1]*bboxes[i,2]/np.power(interaction_length,3))
                ars2.append(bboxes[i,1]*bboxes[i,2]/np.power(interaction_length,2))
        color_palette = plt.cm.plasma
        rng = [0, 2]
        rng2 = [0, 2]
        rng2d = [rng, rng]
        plt.rcParams["font.family"] = "Times New Roman"
        plt.figure(1, figsize=[11.82,5.91])
        plt.subplot(121,ylabel="Threshold for " +str(j)+"D framework", xlabel="Organic cation volume/int.l.$^3$")
        plt.gca().set_title("Organic cation volume vs. " + str(j) + "-dimensional threshold, N = " + str(len(dims)))
        plt.hist2d(ars1,dims,density=True, range=[[0,10], [0,4]],bins=[20,20], cmap=color_palette)
        plt.subplot(122,ylabel="Threshold for " +str(j)+"D framework", xlabel="Minimal face area/int.l.$^3$")
        plt.gca().set_title("Minimal face area vs. " + str(j) + "-dimensional threshold")
        plt.hist2d(ars2,dims,density=True, range=[[0,4], [0,4]],bins=[20,20], cmap=color_palette)
        #plt.subplot(111,ylabel="No. of structures", xlabel="Area/(int. l.)$^2$")
        #plt.gca().set_title("Bounding box minimal face area for " + str(j) + "D structures")
        #plt.hist(ars4,bins=20,range=[0,4])
        plt.savefig(figdir + "Figure_depnd_"+str(j)+".png")
        plt.clf()

if __name__=="__main__":

    plot_all()
```

Bounding box calculations

For determining the dependence of cation geometry on the dimensionality of the inorganic framework, we have extracted the crystal structures of HOIHs present in CCDC crystal structure database,[7] selected by containing at least one M-X bond and not containing any M-Q bonds (where Q = B, C, N, O, Si, P, S, As, Se, Te), resulting in 13773 unique crystal structures with 3D coordinates determined. For each structure, the dimensionality for $t_{\mathrm{n}} = 1$ was calculated. Moreover, the organic cation bounding box was calculated using CSD Python API.[8]

Python code for bounding box calculation

```
#dependencies: ccdc, tqdm
from ccdc import io
from ccdc.descriptors import MolecularDescriptors
from tqdm import tqdm
from global_vars import DIR, target_entries
#everything is contained in ccdc libraries, this just dumps everything in one file
csd_entry_reader = io.EntryReader(target_entries)
with open(DIR+"bb.vectors", "w") as f:
    for entry in tqdm(csd_entry_reader):
        f.write(entry.identifier+"#")
        a = 0
        b = 0
        c = 0
        vol = 0
        for mol in entry.molecule.components:
            try:
                if mol.is_organic:
                    box  = MolecularDescriptors.PrincipleAxesAlignedBox(mol)
                    if box.volume>vol:
                        vol = box.volume
                        a = box.x_vector.length
                        b = box.y_vector.length
                        c = box.z_vector.length
            except:
                pass
        f.write(str(a) + "," +str(b) + "," + str(c)+"#")
        f.write("\n")
```

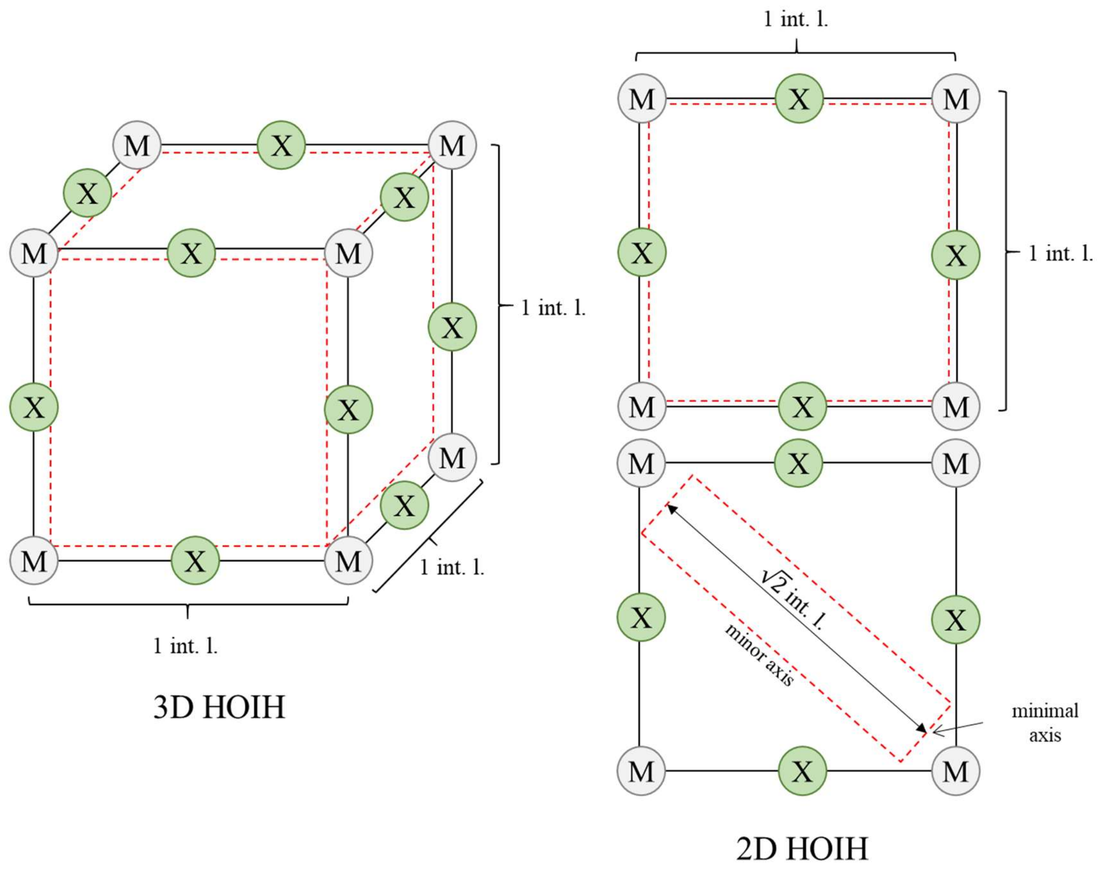

Figure S12. Theoretical limits of the organic cation bounding box (red lines) for 3D HOIHs (isometric view) and 2D HOIHs (top view).

References

(1) Delgado-Friedrichs, O.; O'Keeffe, M. Identification of and Symmetry Computation for Crystal Nets. *Acta Crystallogr A* **2003**, *59* (4), 351–360. https://doi.org/10.1107/S0108767303012017.

(2) Blatov, V. A.; Shevchenko, A. P.; Proserpio, D. M. Applied Topological Analysis of Crystal Structures with the Program Package ToposPro. *Cryst Growth Des* **2014**, *14* (7), 3576–3586. https://doi.org/10.1021/cg500498k.

(3) Gao, H.; Wang, J.; Guo, Z.; Sun, J. Determining Dimensionalities and Multiplicities of Crystal Nets. *NPJ Comput Mater* **2020**, *6* (1), 143. https://doi.org/10.1038/s41524-020-00409-0.

(4) Mounet, N.; Gibertini, M.; Schwaller, P.; Campi, D.; Merkys, A.; Marrazzo, A.; Sohier, T.; Castelli, I. E.; Cepellotti, A.; Pizzi, G.; Marzari, N. Two-Dimensional Materials from High-Throughput Computational Exfoliation of Experimentally Known Compounds. *Nat Nanotechnol* **2018**, *13* (3), 246–252. https://doi.org/10.1038/s41565-017-0035-5.

(5) Ashton, M.; Paul, J.; Sinnott, S. B.; Hennig, R. G. Topology-Scaling Identification of Layered Solids and Stable Exfoliated 2D Materials. *Phys Rev Lett* **2017**, *118* (10), 106101. https://doi.org/10.1103/PhysRevLett.118.106101.

(6) Shannon, R. D. Revised Effective Ionic Radii and Systematic Studies of Interatomic Distances in Halides and Chalcogenides. *Acta Crystallographica Section A* **1976**, *32* (5), 751–767. https://doi.org/10.1107/S0567739476001551.

(7) CSD 5.43 with updates up to September 2022.

(8) Groom, C. R.; Bruno, I. J.; Lightfoot, M. P.; Ward, S. C. The Cambridge Structural Database. *Acta Crystallogr B Struct Sci Cryst Eng Mater* **2016**, *72* (2), 171–179. https://doi.org/10.1107/S2052520616003954.
